# Improving the Longevity of Li-Mediated Ammonia Synthesis via Pulsed Electrolysis under High Current Densities

**DOI:** 10.1021/acsami.6c04372

**Published:** 2026-06-02

**Authors:** Ojo Friday Abraham, Brenden M. Arndt, Reza Nazemi

**Affiliations:** † School of Materials Science and Engineering, 3447 Colorado State University, Fort Collins, Colorado 80524, United States; ‡ Department of Mechanical Engineering, Colorado State University, Fort Collins, Colorado 80523, United States

**Keywords:** lithium-mediated ammonia synthesis, high current density, electrode recycling, pulsed electrolysis, solid electrolyte interphase, dynamic interfacial control, electrode durability, porous gas diffusion electrode

## Abstract

Ammonia synthesis via lithium-mediated nitrogen reduction reaction (Li-NRR) has emerged as a promising electrochemical alternative to the Haber–Bosch process, enabling decentralized, sustainable ammonia production under ambient conditions. Despite this, barriers linger in advancing its efficiency, reaction kinetics, and sustainable operational stability. In this context, we present a high-performance Li-NRR system characterized by a recyclable NiCu_1_Ru_100h_ foam cathode and a pulsed-voltage operation within a single-cell reactor configuration. Constructed from a Ni foam coated with Cu and Ru, the NiCu_1_Ru_100_ electrode presents a large active surface area and catalytic spots, enabling strong lithium plating, nitridation, and protonation functions. Under potentiostatic operation at an optimal cathode potential (−12 V vs Ag/Ag^+^), the NiCu_1_Ru_100_ cathode attained an ammonia yield rate approaching ca. 24 nmol s^–1^ cm_geo_^–2^ at a current density of −80.5 mA cm^–2^, alongside a Faradaic efficiency of ca. 9% reaching 20% after constant potentiostatic recycling. The single-compartment cell design, in contrast to a conventional H-cell, indicated that ammonia crossover and anodic ammonia oxidation could substantially influence the apparent performance; we verified this by incorporating an ion-exchange separator and conducting control experiments focused on ammonia oxidation. Significantly, a pulsed operational technique that alternates rapid, intense negative cathodic pulses with anodic stripping pulses markedly reduced the rate of current density loss during recycling of the working electrode, suggesting improved control of solid–electrolyte interphase stability in the Li-NRR process. The cathode was successfully recycled across seven consecutive Li-NRR cycles with minimal structural degradation. This study provides insights into cell configurations and operational strategies that could improve the lifespan and selectivity of lithium-mediated ammonia synthesis.

## Graphical abstract

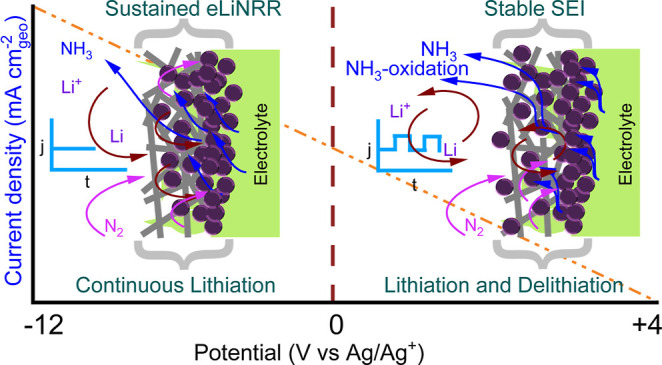

## 1 Introduction

The conversion of nitrogen gas (N_2_) into ammonia under mild working conditions poses a significant scientific challenge with broad implications for sustainability. Among the broad approaches,^1^ the lithium-mediated nitrogen reduction reaction (Li-NRR) has emerged as a rigorously validated technique for the electrochemical synthesis of ammonia under ambient conditions.^2–4^ In this reaction, lithium metal, which is electrodeposited in situ from a lithium salt in a nonaqueous electrolyte, undergoes protonation by a proton source like ethanol of optimized concentration to give ammonia (NH_3_). The lithium-mediated route for NH_3_ synthesis has been successfully demonstrated by using strict N_2_ isotopic controls to eliminate contaminants, confirming that Li in its reduced metallic form has a high affinity for NRR conversion under ambient conditions.^5,6^ However, most of the available reports on Li-mediated NRR are under single-digit or lower current densities,^7–14^ which raises concerns about the reliability of the claimed performances toward commercial applications as these systems are far from industrial competitiveness. To date, only a few studies report double-digit and three-digit current densities,^3,15–20^ with a major drawback in autoclave design to withstand the high pressure of these systems. Aside from operating at high current densities, the need for ambient, decentralized working conditions cannot be overstated.^21^

In this work, we delve into a comprehensive strategy aimed at enhancing the lithium-mediated ammonia synthesis, with a particular emphasis on the critical parameters of cell architecture, electrode material chemistry, and the operational mode. To begin with, we present a novel cathode constructed from a NiCuRu foam, which is essentially a porous nickel scaffold containing copper (Cu) and decorated with ruthenium (Ru). The incorporation of Cu and Ru, which are reported as catalytic metals that plays a crucial role in the dissociation of N_2_ during thermal catalytic processes,^22^ is specifically designed to introduce additional active sites that can significantly facilitate the formation of Li_3_N, with the presence of Cu in the Ni scaffold being instrumental in ensuring optimal electrical conductivity along with a substantial electroplatable surface area. We postulate that this innovative NiCuRu electrode will exhibit both exceptional catalytic activity and remarkable durability, following previous work by our group, with the catalyst synthesis method fully documented,^23^ thereby enabling its repeated use (which we define as recyclability) across an extensive range of LiNRR cycles. In the second part of our investigation, we analyze the influence of the reactor configuration on the overall system performance, comparing a single-cell (SC) design with a conventional H-cell (HC). The limitations of an SC arrangement is such that any NH_3_ that might seep toward the anode has the capability to be converted back into N_2_ gas or nitrogen oxide species (NO_*x*_), which would ultimately undermine the synthesis quantification process we seek to accomplish. We address this critical challenge by integrating a porous Celgard separator into our system and conducting control experiments to elucidate the oxidation of NH_3_ under specific conditions. Finally, we introduce a designed pulsed electrolysis protocol into our experimental framework. Pulsing the cathode between sufficiently negative potentials for Li plating and N_2_ reduction and a moderately positive potential to dissolve excess Li and refresh the electrode surface is expected to suppress continuous solid electrolyte interphase (SEI) growth and minimize the formation of passivation layers over time. Such pulsed or cycled operational methodologies are, to date, relatively uncommon in the existing literature on LiNRR, and our findings indicate that this approach yields significant improvements in both SEI growth control and current density loss during electrode recycling at high current densities. The quasi-reference electrode and the counter electrode were an Ag wire and a Pt coil, respectively, and the electrolyte contained 1 M LiBF_4_ in THF with 0.25% EtOH. Moreover, the novelty of this work entails three advances aimed at improving the practicality of Li-mediated NH_3_ synthesis under ambient pressure: (1) we verified the electrochemical cell architectural effect on NH_3_ oxidation/crossover, (2) we demonstrated a recyclable 3D NiCu_1_Ru_100_ cathode architecture operating at high current densities without a pressurized autoclave, and (3) we implemented a pulsed-voltage protocol that alternates cathodic Li-plating periods with anodic stripping pulses to slow current-density decay and extend electrode recycling cycles.^24^

## 2 Experimental Section

### 2.1 Materials

Tetrahydrofuran (C_4_H_8_O, anhydrous, 99+%, stabilized with BHT preservative), sulfuric acid (H_2_SO_4_, ACS grade), phosphoric acid (H_3_PO_4_, ACS grade), ethanol (anhydrous, 100%, ACS grade) were purchased from Honeywell, Oakwood Chemical, Ward’s Science, and PHARMCO (Greenfield Global), respectively. Lithium tetrafluoroborate (LiBF_4_, 98%), sodium hypochlorite solution (10–15%), sodium nitroprusside dihydrate (99%), ammonium chloride (99.5%), salicylic acid (99%), and sodium hydroxide (97%) were purchased from Sigma-Aldrich. Nickel foam and Celgard membrane were purchased from Fuel Cell Store and CELGARD, respectively.

### 2.2 Electrochemical LiNRR (eLiNRR) Measurements

The electrochemical measurements were carried out in a 3-electrode single-cell (40 mL) (Figure S1A) and an H-cell (60 mL) (Figure S1B) configurations using a Celgard separator at room temperature and ambient pressure. The electrolyte consisted of 1 M LiBF_4_ in 99.75 vol % THF and 0.25 vol % EtOH and was continuously stirred at 160 rpm. The catalyst composition is based on the recently reported performance of Cu_1_Ru_100_ for NH_3_ production by our group, and the detailed synthesis process has been documented.^23^ The working electrode with a geometric area of 1.5 cm^–2^ was prepared by washing the nickel foam in phosphoric acid and thereafter in ethanol to remove organic contaminant before spray-coating the catalyst on both sides (1 mg cm^–2^) and then furnace-drying it at 100 °C for 3 h (Figure S1C). The counter and quasi-reference electrode were a Pt coil and an Ag wire, respectively. The distance between the counter electrode and the working electrode was controlled at around 0.5 cm.

The eLiNRR measurements were carried out with a GAMRY Reference 3000 electrochemical workstation. The activity of our cathode (NFGDE) was evaluated by controlling the applied potential and the reaction time in the electrolyte. The linear sweep voltammetry (LSV) measurements were conducted from 0 to −12 V at a scan rate of 20 mV s^–1^. The area of the working electrode exposed to the electrolyte for eLiNRR was 1.5 cm^2^. Before the eLiNRR measurements, the electrolyte was saturated with N_2_ by purging for 30 min to remove residual air in the system. During the eLiNRR reaction, the reaction system was continuously fed with N_2_ at a bubbling rate of 10 sccm. For comparison purposes, the eLiNRR test was also conducted in Ar-saturated electrolyte solutions under similar concentrations and operating conditions.

### 2.3 Characterizations

The scanning electron microscopy (SEM) images coupled with energy dispersive spectroscopy (EDS) elemental mappings were obtained using JEOL JSM-IT800­(HL) Field Emission SEM. Raman spectroscopy was performed using an XploRA PLUS inverted optical microscope fiber-coupled to a spectrometer (HORIBA Scientific). Stokes and anti-Stokes shifts of a Si wafer were used to calibrate the Raman system, occurring at 521 cm^–1^. All the Raman spectra were acquired under similar ambient conditions.

### 2.4 Ammonia Quantification

The NH_3_ yield in THF samples that have been quenched with deionized water was quantified through the application of the modified indophenol blue spectrophotometric method. Initially, a modest volume of the electrolyte solution of 200 μL was meticulously extracted and subsequently transferred into a 5 mL glass vial, where it was then diluted to a 10-fold volume with ultrapure water to ensure the accuracy of the subsequent measurements. In this step, 200 μL of salicylic acid solution, which possesses a concentration of 1.0 M in a 1.17 M sodium hydroxide (NaOH) solution, was added, followed by the sequential introduction of 50 μL of sodium nitroprusside, prepared at a concentration of 0.1 g in a total volume of 10 mL of water, and 50 μL of sodium hypochlorite (NaClO), characterized by an available chlorine content of 0.35 wt % in a 0.7 M NaOH solution. Following the addition of these reagents, the NH_3_ quantification mixtures were incubated under ambient temperature conditions for a duration of 2 h to facilitate the complete development of the characteristic color, which was subsequently analyzed using a UV–visible spectrophotometer to determine the absorbance values. The calibration curve for the colorimetric quantification of NH_3_ was prepared using various concentrations of NH_4_Cl in the electrolyte solution, similar to the test electrolyte. Prior to subtracting the absorbance of the blank, the absorbance of the NH_4_Cl-stained solutions at 697 nm was plotted as a function of the NH_3_ concentration (Figure S2). For all experiments discussed in this work, the data were closely aligned, with *R* ^2^ typically exceeding 0.99 (Figure S2B,D).

### 2.5 Yield Rate, Faradaic, and Production Energy Efficiency Calculations

The yield rate of NH_3_ was calculated using the following equation

1 $${NH}_{3}yieldrate=C_{NH_{3}}\times V/\left(A_{cathode}\times t\right)$$

Such that $C_{NH_{3}}$ is the measured concentration of NH_3_, *V* is the volume of the test electrolyte, exposed geometric area of the cathode electrode is denoted as *A*_cathode_, and the electrolysis testing time is *t*.

Three electrons are needed to produce one molecule of NH_3_. The NH_3_ production Faradaic efficiency (FE) can be calculated at a given test potential using eq 2.

2 $${NH}_{3}Faradaicefficiency=3\times F\times C_{NH_{3}}\times V/\left(17\times Q\right)$$

Such that the Faraday’s constant (96,485 C mol^–1^) is represented as *F*, the measured NH_3_ concentration as $C_{NH_{3}}$ , the volume of the test electrolyte as *V*, the molecular weight of NH_3_ as 17 g mol^–1^, and the integrated charge from the current–time curve as *Q*.

The production energy efficiency (PEE) can be calculated using eq 3.

3 $$PEE\;\left(\%\right)=\frac{\Delta G\text{for}ammoniageneration\times ammonia\;generated\;\left(mol\right)}{\int IVdt}\times 100$$

where the NH_3_ generation free energy (Δ*G*) is 339 kJ mol^–1^ and ∫*IV* d*t* (J) is the consumed electricity in the process, also referred to as the energy input, and the full cell potential is denoted as V.

## 3 Results and Discussion

### 3.1 Synthesis and Characterization of Catalyst

The morphological and compositional characteristics of the 3D NiCu_1_Ru_100_ porous gas diffusion electrode (GDE) shows a uniform spray-coated Cu_1_Ru_100_ catalyst (1 mg/cm^2^) on the nickel foam substrate (Figure 1). The porous cathode (Figures 1A and S1C) 3D architecture affords a substantial surface area with a cut-out geometrical surface area of 1.5 cm^2^. We used this electrode, with an adequate void volume, in electrochemical tests to support lithium intercalation, reduction, nitridation, and protonation for NRR, while also facilitating electrolyte infiltration and reducing lithium migration during electrolysis. This structural morphology has been leveraged as a conductive framework in Li-NRR systems to promote uniform Li reduction and deposition, thereby mitigating localized current hotspots that can lead to premature passivation and eventual cathode deactivation.^12,19,25,26^ The interconnected porosity observed in this work is therefore advantageous for sustaining high current densities in the double- and triple-digit mA/cm^2^ range, supporting industrially relevant operation during Li-NRR, particularly under the pulsed electrolysis conditions employed in this study.

**Figure 1:**
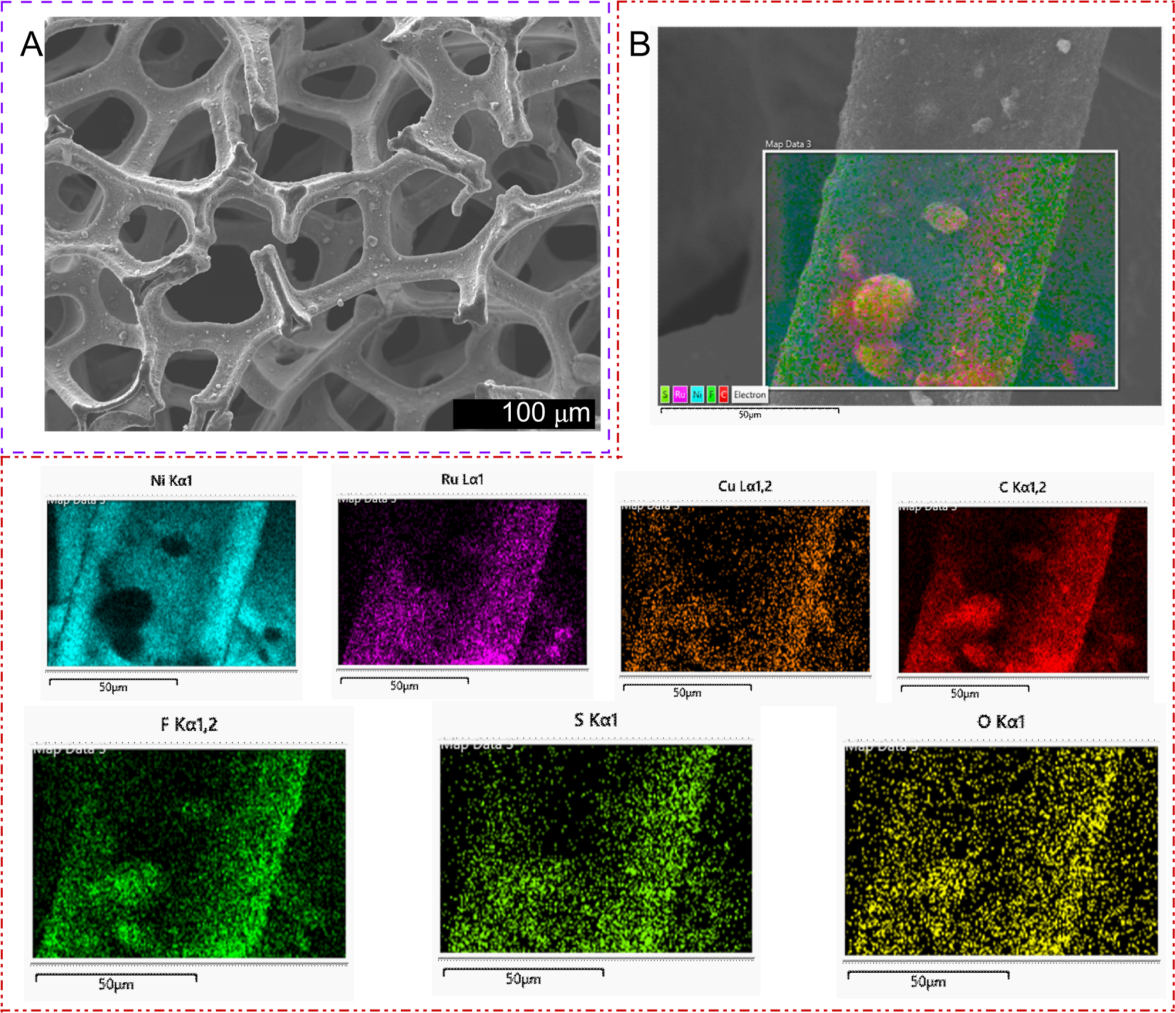
(A) SEM image of NiCu_1_Ru_100_ before lithium mediation, and (B) EDX elemental mappings of synthesized catalyst, Cu_1_Ru_100_, spray-coated (1 mg/cm^2^) on nickel foam.

The EDX mapping verifies the balanced dispersion of the Cu_1_Ru_100_ catalyst on the nickel foam obtained from the corresponding SEM image (Figure 1B). A uniform catalytic distribution is paramount for stabilizing Li plating and reducing the formation of high-resistance SEI, thereby improving the overall performance of Li-NRR.^27^ The detection of oxygen signals in the EDX map aligns with the presence of a partial oxide layer commonly observed on nickel foam gas diffusion electrodes (NFGDEs) and transition-metal coatings. Notably, the presence of oxygen is not unexpected and often plays an advantageous role in modulating the interfacial chemistry of NFGDEs. It was demonstrated that trace amounts of oxygen species can adjust the SEI composition, thereby preventing excessive accumulation of lithium hydride (LiH) or lithium oxide (Li_2_O) and enhancing Faradaic efficiency.^28^

The uniform distribution of ruthenium (Ru) (Figure 1B) is of particular significance. Recent studies indicated that Ru can enhance catalytic robustness^25^ by altering the electron density and localized surface adsorption environment at the Li-electrode interphase, thereby facilitating stable Li-NRR and enabling effective nitridation.^29–31^ Within the aim of this work to enhance electrode longevity under high-current Li-NRR conditions, such uniform incorporation of a trimetallic scaffold is crucial with the smooth and uniformly distributed NiCu_1_Ru_100_ GDE (Figure 1A) providing a stable architecture for the pulsed electrolysis strategy proposed in this work.

The SEM and EDX analyses convey that the NiCu_1_Ru_100_ GDE possesses the requisite structural and compositional characteristics as expected for the experimental pathways inherent for the Li-NRR system. The morphology of the electrode and the chemistry at the interphase exert a profound influence on the long-term performance of Li-NRR, oftentimes superseding the intrinsic catalytic properties alone.^31^ The precisely defined NFGDE architecture and uniform catalytic dispersion presented in this study align with these design principles and facilitate the enhanced stability observed during pulsed electrolysis at high current densities.

### 3.2 Ammonia Oxidation and Crossover Verifications

Another important verification prior to focusing on a specific electrochemical cell configuration is the potential for ammonia oxidation, given the limited studies addressing this possibility in single-cell, nonaqueous Li-mediated NRR systems (Figure S1). As most available reports do not simulate identical cell configurations and electrolyte chemistry, we address this by conducting a comparative analysis of electrochemical performance and ammonia yield determined via the indophenol blue chromogenic reaction.^5,7^ The absorbance at the 695 nm was used to establish the calibration curve (Figure S2) and the NFGDE foam in both single-cell and H-cell/Celgard membrane configurations under the conditions of a similar charge equivalent to ca. 400 C for the Li-NRR (Figure 2) used for this comparative study. The single-cell configuration yields a markedly superior NH_3_ yield rate of ca. 1.5 mg h^–1^ cm^–2^ in comparison to the H-cell configuration (0.5 mg h^–1^ cm^–2^) (Figure 2A). This enhancement is consistent with the reduced ohmic resistance and reduced interfacial transport limitations typically observed in single-compartment Li-NRR systems.^32^ This pivotal factor is the close integration of Li plating, N_2_ diffusion, and the availability of protons, and these are more uniformly facilitated with the geometries of the single-cell architectures.

**Figure 2:**
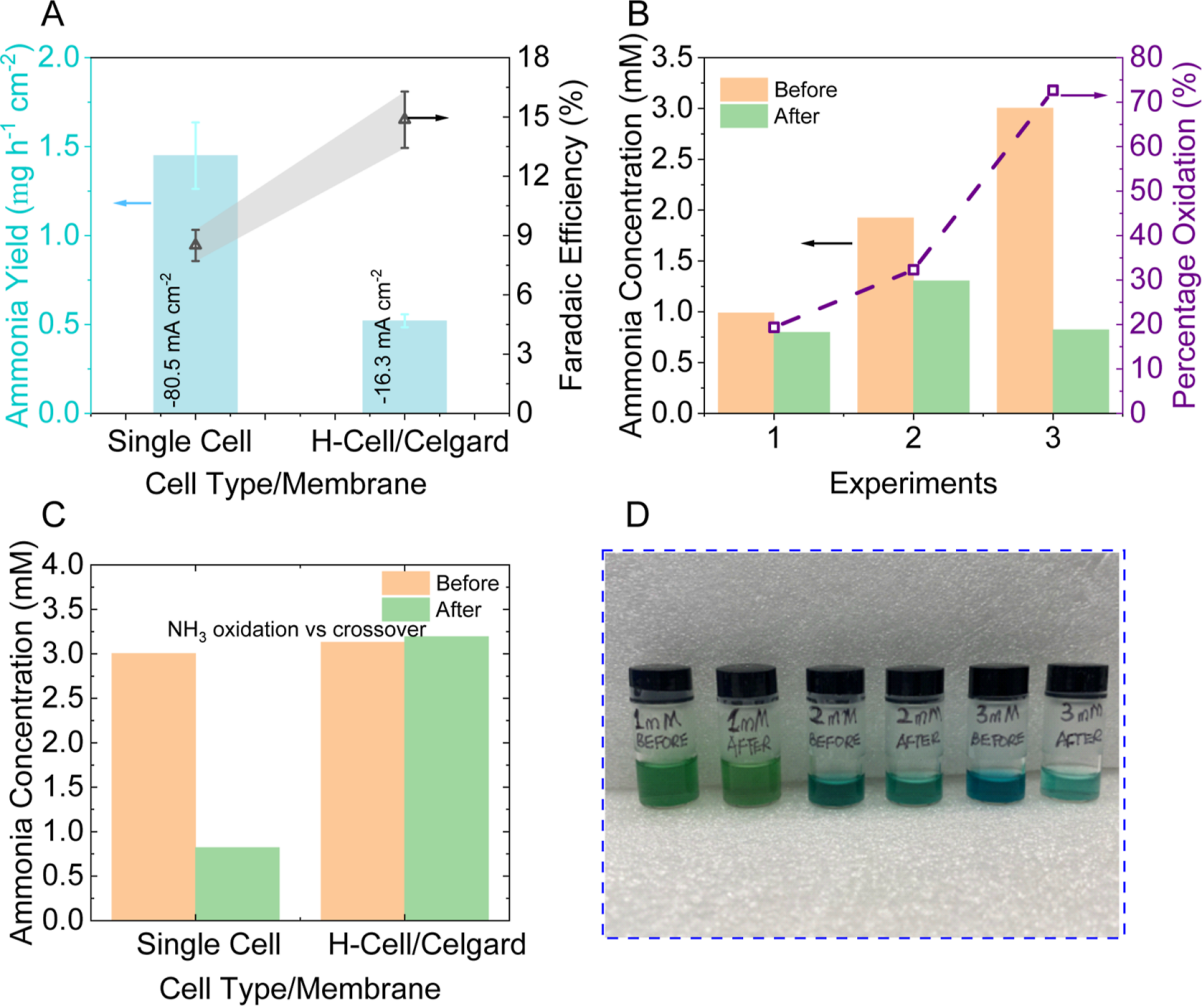
(A) NH_3_ yield rates and Faradaic efficiencies of electrocatalytic Li-NRR NiCu_1_Ru_100_ foam cathode under a comparable charge of 400 C for single-cell and H-cell configurations, mean ± SD, *n* = 3. (B) Ammonia oxidation behavior in a single cell at a comparable charge of 400 C with the actual electrocatalytic Li-NRR potentiostatic experiments. (C) Ammonia oxidation and crossover comparison as a function of electrochemical cell geometries, and (D) pictorial view of samples before and after NH_3_ oxidation experiment in a single-cell configuration.

The possible NH_3_ oxidation in the single-cell on account of electrode proximity was evaluated at increasing known concentrations of NH_3_ following continuous purging with Ar gas to eliminate any interference of any NH_3_ forming species under ca. −80 mA cm^–2^ for 1 h to establish the trend in percentage loss of NH_3_ (Figure 2B). A marked decrease in NH_3_ concentration after potentiostatic testing within the cell was recorded, thereby validating that there is a possible loss of NH_3_ in the single-cell, and this could lead to underestimation of recorded yield and FE. This is further supported in the H-cell, as there was no evident decrease in the NH_3_ concentration after potentiostatic testing under similar charge in the cell. We believe this will eventually address the question of whether or not there is NH_3_ oxidation in acidic electrolytes.^3^ We will recommend that caution be taken to make sure the electrolyte is not excessively diluted to avoid transition to an aqueous system, as we observe a slightly linear trend in the percentage of oxidation with NH_3_ concentration (Figure 2B). Compared with the single cell (Figure 2C), the H-cell configuration exhibits markedly little or no changes in the NH_3_ concentration before and after potentiostatic testing, thereby supporting the hypothesis that membrane-separated cells can mitigate crossovers but often at the expense of diminished reaction rates due to ohmic loss and mass transport constraints.^32^ The visual inspection of the electrolyte samples prior to following NH_3_ oxidation in the single-cell configuration affirms the colorimetric alterations associated with ammonia loss (Figure 2D). The notable color change observed postoxidation is consistent with the ammonia quantification (Figure 2B) and serves to establish that although single-cell configuration results in higher NH_3_ yield rate on account of higher mass transport, proton availability, and electrode proximity, it is susceptible to a level of NH_3_ oxidation at the anode, and this can lead to underestimation of overall process performance. Given the industrial advantage of reduced ohmic losses in the single-cell configuration, which outweighs potential NH_3_ losses from oxidation, we evaluated the durability of NFGDEs at high current densities using constant and pulsed potentiostatic modes to validate overall performance. In this study, we did not perform quantitative isotope labeling measurements using ^15^N_2_, as such measurements were rigorously performed on the catalyst for ammonia synthesis in our prior study.^23^ Moreover, under the test conditions, especially for the single cell configuration at −12 V (−80.5 mA cm^–2^), an average of 127 μmol ± 14 μmol (2.2 mg ± 0.2 mg) ammonia was produced from three independent measurements with an average Faradaic efficiency of 8.5% ± 0.7%, which is orders of magnitude higher than the false positive ammonia production from various sources of contamination.^5,33,34^ In the current study, several precautions were additionally implemented to minimize the possibility of false-positive ammonia detection, including the use of high-purity gases, a fully enclosed electrochemical setup, controlled operation within a fume hood environment, Ar control experiments, and independent replicate measurements.

### 3.3 Electrochemical Tests

In order to operate the NFGDEs under high current densities, the electrocatalytic performance was evaluated across a spectrum of applied potentials of −8, −10, −12, −14, and −16 V (Figure 3). This comprehensive analysis not only establishes a foundational understanding of the electrochemical characteristics of the system prior to pulsed electrolysis but also underscores the sensitivity of Li-NRR to variations in the applied potential.

**Figure 3:**
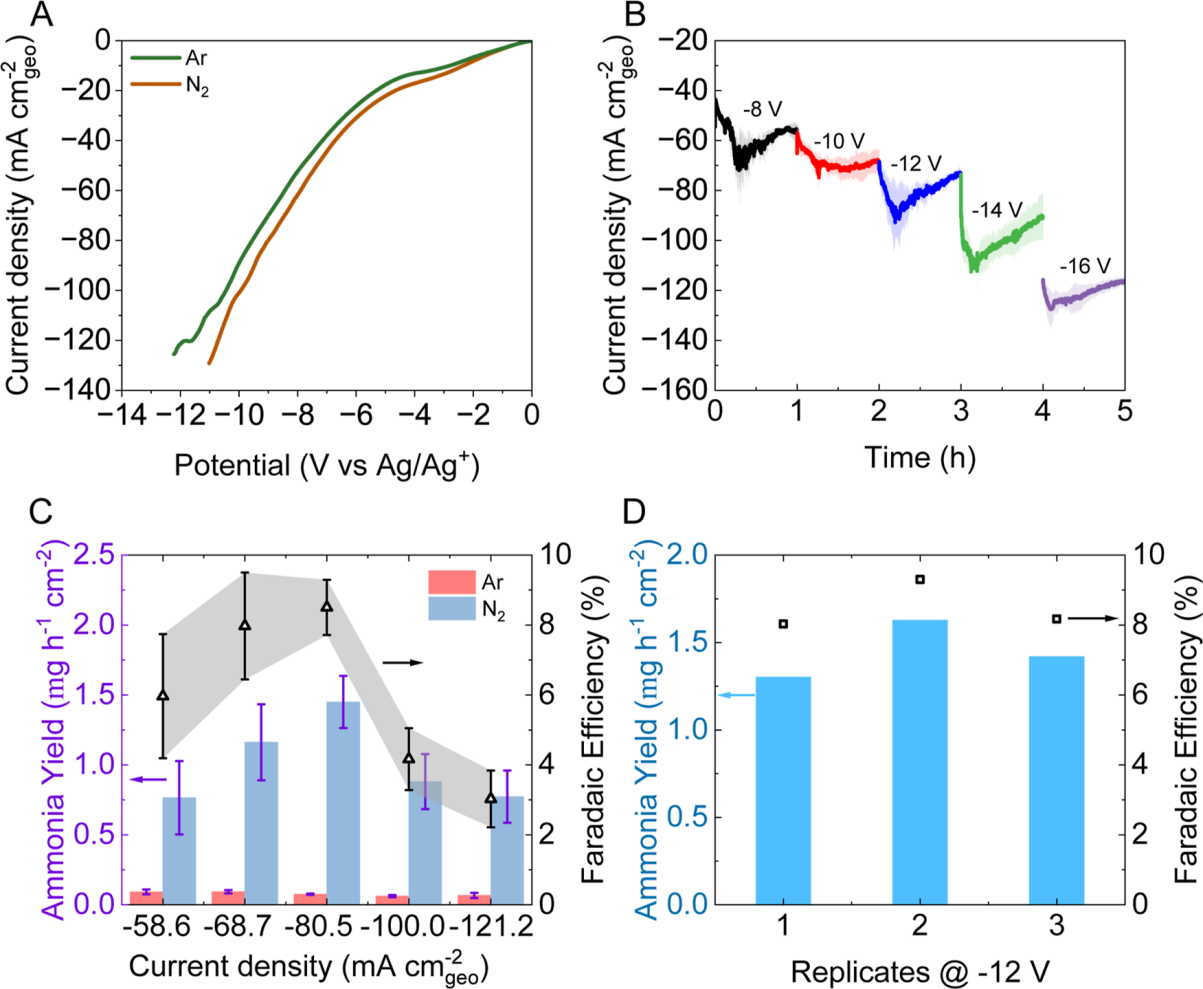
(A) Linear sweep voltammetry curves of the NiCu_1_Ru_100_ electrode under N_2_ and Ar atmosphere. (B) Current–time profiles of NiCu_1_Ru_100_ electrode at various applied potentials in 1 h electrolysis test, mean ± SD, *n* = 3. (C) NH_3_ yield rates and Faradaic efficiencies of electrocatalytic Li-NRR at corresponding current densities for applied potentials of −8, −10, −12, −14, and −16 V, mean ± SD, *n* = 3. (D) Replicates for electrocatalytic Li-NRR at a potential of −12 V.

The linear sweep voltammetry (LSV) curves under saturated gases (Ar and N_2_) of the NFGDEs (Figure 3A) depict that the current response under the nitrogen saturated condition increasingly diverges from that observed under argon, particularly at potentials that fall below −10 V relative to the pseudo reference (Ag/Ag^+^) electrode, which serves as a critical indicator of the onset of lithium plating that is concurrently associated with the activation of N_2_ gas. This comparable divergence under the Ar and N_2_ in terms of current density response aligns with a previously conducted mechanistic investigation that has demonstrated that the reduction of Li is the rate-determining step in the formation of Li_*x*_N intermediates instead of N_2_ reduction, which are of paramount importance for the dissociative Li-mediated reduction pathway of N_2_.^7^ The current–time profiles (Figure 3B) under a potential spectrum of −8 V to −16 V show a linear increasing current density behavior. Increasing current densities in response to increasing negative voltage perturbation is indicative of the anticipated rise in the rate at which Li plating occurs, a critical process in eLi-NRR systems. Nevertheless, the rapid decay of current densities at potentials beyond −12 V highlights the inherent instability of the electrode–electrolyte interface under continuous lithiation at elevated reduction potentials.^35^ A behavior consistent with the challenges that have been well-reported in the Li-mediated studies on the effect of SEI on the activity and selectivity toward Li-NRR. The phenomenon of excessive Li deposition tends to promote the growth of SEI, accelerate electrolyte decomposition, and passivate the active sites on the electrode.^36^ These degradation processes are of paramount importance, as they directly contribute to a reduction in Faradaic efficiency (FE), thereby providing a compelling rationale for the explanation of the pulsed-electrolysis strategy that will be elaborated in subsequent sections.

Ammonia yield was quantitatively assessed via the modified indophenol method under saturated Ar and N_2_ conditions to avoid false positives from the prepared catalyst itself and gaseous sources (Figure 3C). Experiments were conducted under controlled ambient conditions in a fume hood. The production of NH_3_ experiences a notable increase, reaching its peak at ca. −80 mA cm^–2^ under an applied potential of −12 V, thereby establishing the optimal operational window for the Li-NRR as facilitated on the NFGDEs. Beyond −12 V results in a decline in NH_3_ yield, despite increasing current densities, indicating a transition of the system from active LiNRR to various side reactions at higher overpotentials. This phenomenon is often described as a volcano-shaped dependence of ammonia yield on current density, with the decline in yield attributed to mass-transport limitations and SEI overgrowth under high cathodic-bias conditions.^35^ Moreover, the observed insignificant NH_3_ signal in the presence of saturated Ar serves to support that the NH_3_ yield is a consequence of LiNRR processes rather than from contaminations or the decomposition of the electrolyte itself. Replicate experiments at an applied voltage of −12 V demonstrate the reproducibility with respect to both yield and the FE achieved throughout these trials, as well as across the test voltage spectrum when also compared with bare nickel foam without catalyst staining (Figures 3D and S3). The remarkably narrow distribution of the measured values confirms the stable availability of N_2_ gas and indicates consistent behavior in Li plating, alongside reliable and precise quantification procedures that constitute a thorough and rigorous benchmarking process for LiNRR.^5^ This observed reproducibility not only emphasizes the inherent robustness of the NFGDEs architectural framework but also establishes a solid baseline against which the subsequent effects of pulsed electrolysis will be evaluated and analyzed under optimum cathodic voltage of −12 V.

While simultaneously establishing the rationale and motivation for the pulsed electrolysis strategy, we conducted and reported a comprehensive mechanistic understanding of the various degradation pathways that the NFGDEs undergo during the LiNRR process (Figure 4). The integrated data obtained from Raman spectroscopy and electrochemical impedance spectroscopy (EIS) elucidate the intricate chemical and interfacial transformations that occur during extended operational periods, particularly under elevated cathodic potentials, which significantly influence the performance and longevity of the electrode material.

**Figure 4:**
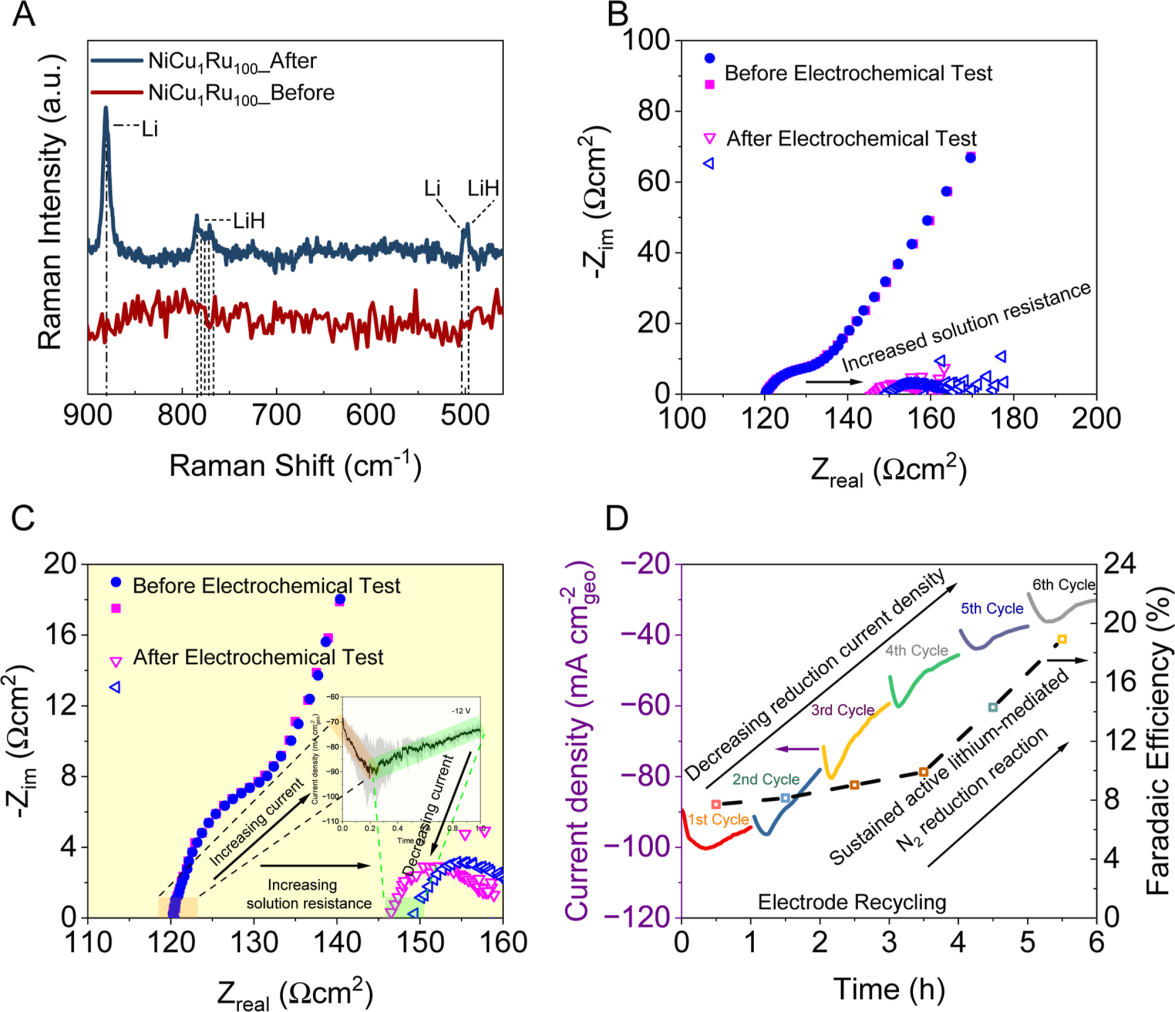
(A) Raman spectra of test electrode NiCu_1_Ru_100_ before and after electrocatalytic Li-NRR. (B) Electrochemical impedance spectroscopy Nyquist for before and after electrochemical Li-NRR of NiCu_1_Ru_100_ test electrode. (C) Electrochemical impedance spectroscopy (200 kHz to 1 Hz frequency range, 10 mV amplitude) measured at open circuit potential. The data obtained with the NFGDEs in 1 M LiBF_4_ + THF with 0.25 vol % EtOH shows the effect of solution resistance changes on the current–time (inset) behavior and (D) current–time curves and Faradaic efficiencies of the potentiostatic recycled NiCu_1_Ru_100_ electrode under −12 V applied potential.

The Raman spectra for the NFGDEs before and after LiNRR show distinct vibrational bands within the spectra range of ca. 850 to 880 cm^–1^ and 500 to 790 cm^–1^, which are indicative of the presence of lithium (Li) and lithium hydride (LiH) species, respectively (Figure 4A).^37^ The detection of these unique signals after LiNRR functions as convincing proof for the reduction of Li^+^ to Li and LiH constituents that are situated in the complex chemistry of the SEI. It is important to note that these inorganic passivation products have been reported to increase interfacial resistance and to deactivate electrodes in systems associated with LiNRR.^36,38^ The accumulation of such species directly impedes the transport of Li ions and simultaneously obstructs the formation of reactive lithium nitride (Li_*x*_N) intermediates, which are essential for the synthesis of ammonia. This spectroscopic evidence confirms that the NFGDE undergoes continuous, progressive modification of its surface chemistry over extended operational cycles, underscoring the critical need for regeneration mechanisms, such as anodic pulse stripping, to restore its functionality. The electrochemical characteristics of the NFGDEs pre- and post-LiNRR further indicate features arising from the SEI formation (Figure 4B,C). It is noteworthy that a significant increase in both the intercept observed at high frequencies and the diameter of the semicircle is observed following the electrochemical LiNRR, indicating a substantial increase in both solution resistance and charge-transfer resistance within the system. This specific behavior closely aligns with reported SEI effects, electrolyte degradation, and blockage of the working cathode surface on solution and charge-transfer resistances as a function of electrolyte salt and proton source concentrations.^14,39^ These parameters are suggested to contribute to the instability in the LiNRR process in this work. To support this effect further, the inset (Figure 4C) relates the relationship between the rising solution resistance and the concomitant reduction in the reduction current density magnitude that occurs during the operation under constant potentiostatic conditions. Uncontrolled Li plating can lead to rapid instability in interfacial conductivity due to the relationship between SEI evolution and the decline in electrochemical performance.

The recyclability of the NFGDEs under constant potentiostatic conditions of −12 V was investigated to establish the effect of continuous SEI buildup and previously plated Li on the yield of NH_3_, FE, and overall performance (Figure 4D). The current–time profiles, which were recorded over a series of six consecutive cycles, reveal a discernible and progressive decrease in current density, thereby providing empirical confirmation of the cumulative detrimental effects that were previously inferred from the Raman spectroscopy (Figure 4A) and EIS data (Figure 4C). However, the FE shows an increasing trend with increasing cycles, which clearly indicates a maintained selectivity for NRR using the plated Li, even under a loss of current density. These observations align with and are corroborated by recent reports of SEI overgrowth, which reduces the active surface area and impairs pathways for continuous conductivity.^14,24^ These pivotal findings lay the groundwork for the application of anodic pulses to alleviate the accumulation of SEI effectively and, in turn, restore the electrochemical activity of the electrode, thereby enhancing electrical conductivity over multiple cycles.

The retention of current over extended cycling of an intermittent resting time of 5 min of the NFGDEs under pulsed electrolysis serves to assess the effect of lithiation and delithiation on the SEI stability for longevity of current densities of the electrodes beyond constant potentiostatic conditions (Figure 5). Application of intermittent anodic perturbations plays a critical role in significantly alleviating the current degradation that is typically observed during the conventional constant potentiostatic cycling (Figure 4D), thereby leading to enhancement in the operational longevity of the system. Pulsing cycles consisting of cathodic (−12 V) and anodic (+4 V) with the cathodic and anodic pulses of 1 min each were first adopted before testing at a slightly lower anodic potential (+3) than the reduction potential of Li (Figure 5A). Throughout each cycle, the cathodic current profile exhibits a discernible, gradual decline in magnitude. This phenomenon is indicative of SEI formation and a corresponding partial passivation of the NFGDE surface. Notably, the ensuing anodic step partially restores the interface’s accessibility for subsequent electrochemical processes. This is supported by the relatively modest current-density losses observed over the initial five cycles, with a differential range of 17% to 26%. When compared with the continuous potentiostatic case (Figure 4D), these findings underscore the pronounced ability of anodic pulses to effectively control the surface layers formed by SEI buildup and electrolyte decomposition, which are detrimental to the long-term efficiency of LiNRR. To further establish the effect of pulsing time and anodic potential on the NFGDE stability and current density losses, we tested a cathodic (−12 V) pulse time of 10 s and an anodic (+3 V) pulse time of 1 s (Figure 5B). Despite the relatively brief anodic phase, the observable regeneration effect remains pronounced across the initial three cycles, as evidenced by performance changes of 18% and 32%. However, during the progression into the fourth and fifth cycles, a marked acceleration in current density loss is observed, with values ranging from 50% to 62%, which suggests that this anodic pulse fails to adequately disrupt the growth of the SEI once the formation of a dense passivation layer has occurred (Figure S6). This observed behavior aligns with mechanistic analyses that demonstrated that the restructuring of the SEI and the oxidation of the plated Li to Li^1+^ requires a slightly higher amount of anodic charge transfer than the reduction potential of Li (−3.1 V) to effectively remove the accumulated lithium-based species present in the electrode–electrolyte interface. Moreover, it is important to emphasize that even the implementation of the short-pulse protocol surpasses the performance achieved with constant potentiostatic recycling in terms of the severity of current density losses.

**Figure 5:**
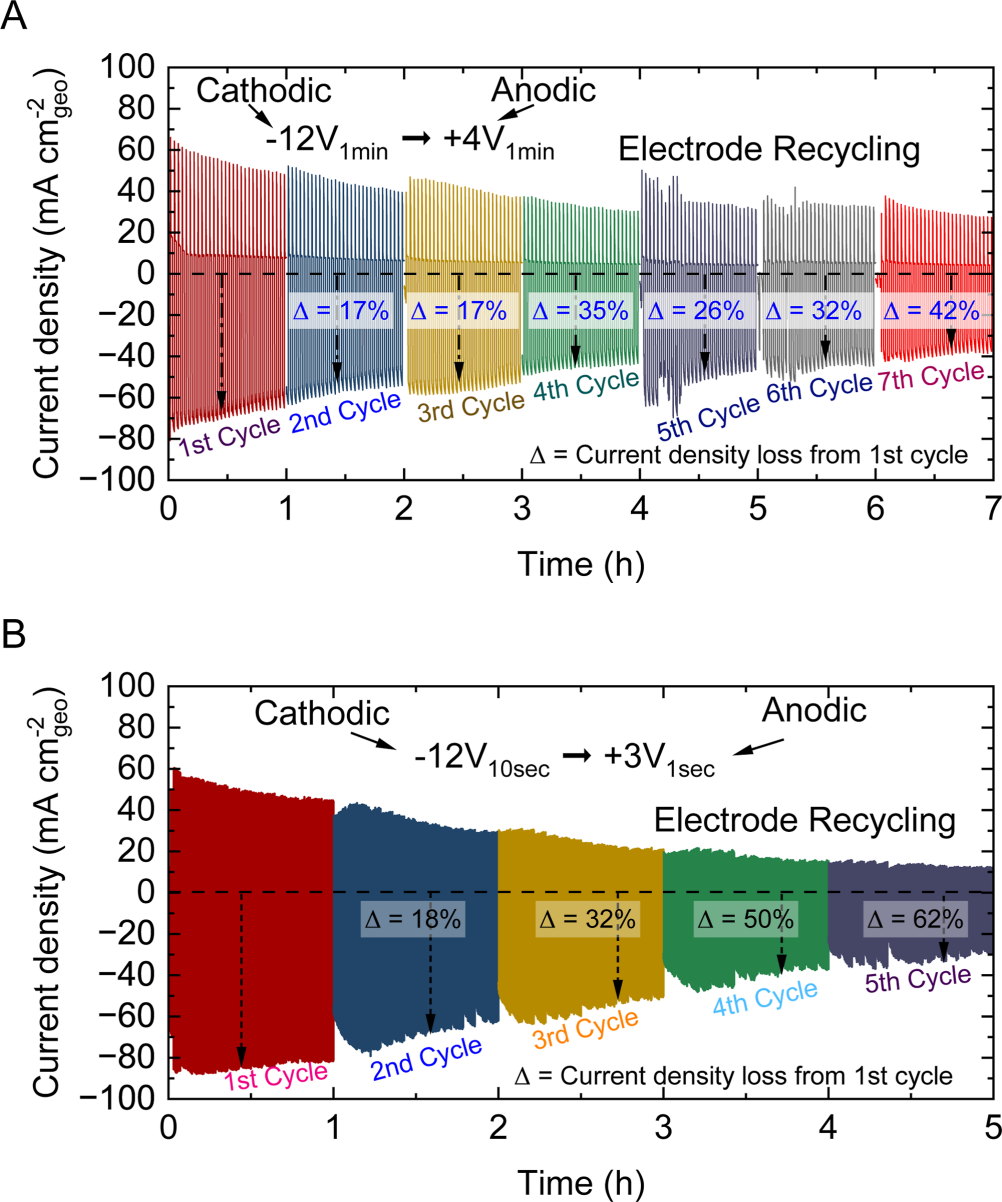
Pulsing cycles of recycled NiCu_1_Ru_100_ electrode under pulsing conditions of (A) −12 V for 1 min and +4 V for 1 min for a total duration of 1 h, and (B) −12 V for 10 s and +3 V for 1 s for a total duration of 1 h.

It turns out there is a correlation between the longevity of the electrode and the parameters of pulsing and the amount of recycling cycles before substantial loss in activity of the NFGDEs (Figure 5). The implementation of longer anodic pulses facilitates a more efficient removal of passivation layers (Figure S6), whereas the utilization of shorter pulses, while providing some degree of mitigation against degradation, ultimately leads to a deterioration of the interfacial electrical conductivity over time (Figure 5B). The implications of these findings are consistent with the contemporary methodologies that focus on electrolyte engineering,^8^ the fine-tuning of oxygen-based SEIs,^28^ and the exploration of alternative proton donors,^40^ all of which are strategically designed to mitigate the effects of irreversible passivation that adversely affects electrode performance. Therefore, pulsed electrolysis emerges as a methodologically coherent and operationally straightforward strategy with significant promise for prolonging electrode lifetimes, particularly under the demanding conditions of high-current operation in LiNRR applications.

Mechanistically, constant cathodic bias drives continuous Li plating and electrolyte decomposition, thickening the SEI and steadily increasing charge-transfer and mass-transport resistances. The anodic pulses implemented here act as controlled stripping steps that (i) dissolve excess plated Li (including electronically passivated Li), (ii) partially remove or restructure passivating Li-containing interphase products, and (iii) reopen pore volume/active area for subsequent lithiation–nitridation–protonation. This interpretation is consistent with prior Li-NRR studies emphasizing SEI-governed selectivity/stability and with pulse-plating literature in Li–metal systems, where pulsed waveforms and rest periods reduce dendrite growth by enabling Li^+^ redistribution and mitigating local current hotspots.^41^ In our system, this interphase-renewal concept is consistent with the improved current-density retention observed under pulsed protocols (Figure 5) relative to constant potentiostatic recycling (Figure 6).

**Figure 6:**
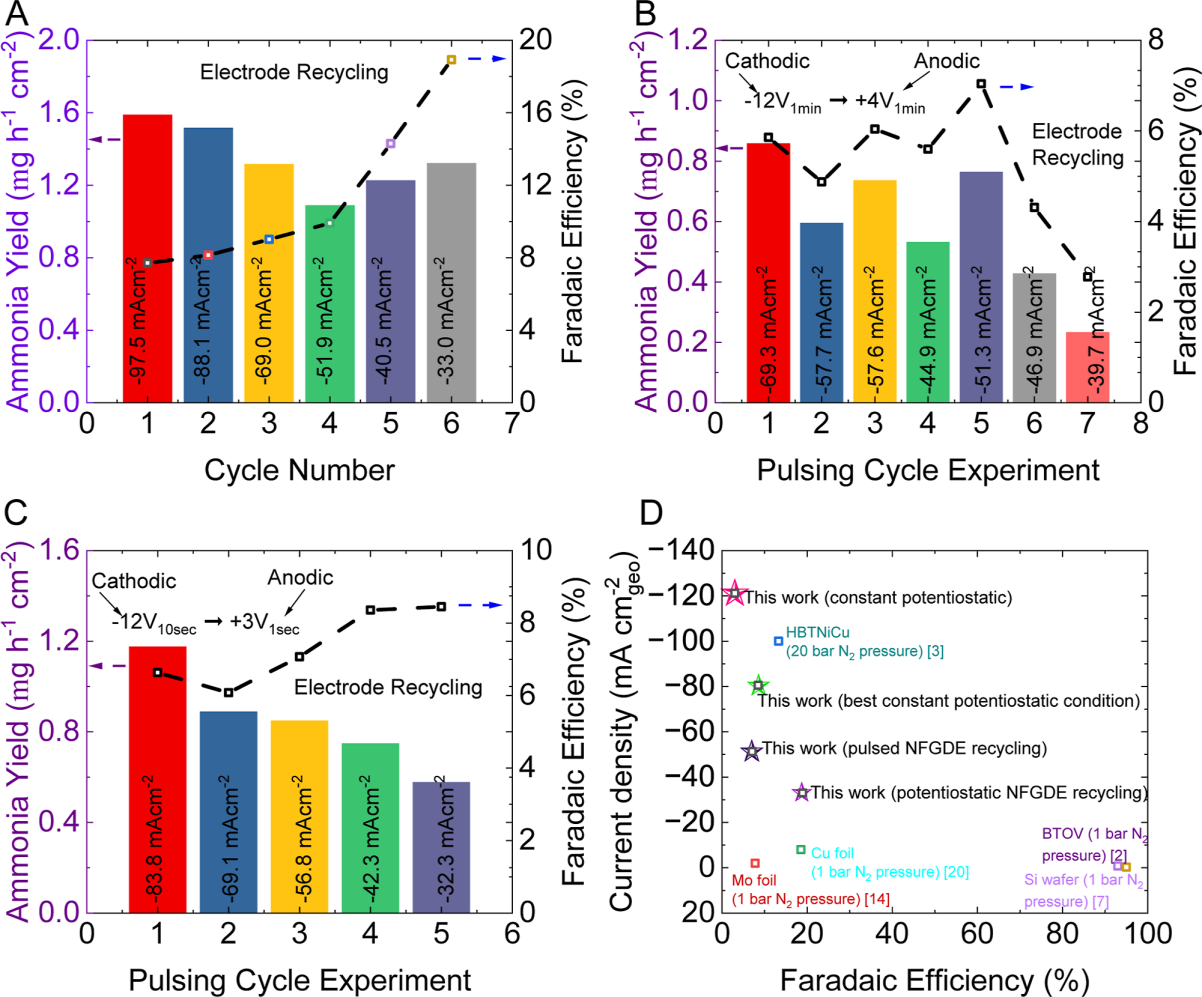
NH_3_ yield rates and Faradaic efficiencies for the recycled electrode at −12 V potential under (A) potentiostatic perturbation, (B) pulsing perturbation at −12 V for 1 min and +4 V for 1 min for a total duration of 1 h, (C) pulsing perturbation at −12 V for 10 s and +3 V for 1 s for a total duration of 1 h, and (D) current densities and Faradaic efficiencies of prior studies on Li-mediated ammonia synthesis with different working electrodes and operating pressure, and their comparison with this work (Table S1).

The implementation of pulsed electrolysis not only markedly enhances the current density longevity of the NFGDEs but also serves to stabilize the SEI under conditions of operation characterized by high current densities (Figures 6 and S6). The activity and selectivity toward LiNRR by the NFGDE recycling was carried out under constant potentiostatic at −12 V (Figure 6A). Throughout the course of these cycles, ammonia production rate exhibits a U-shaped behavior, diminishing from ca. 1.6 mg h^–1^ cm^–2^ during cycle 1 to about 1.3 mg h^–1^ cm^–2^ by the time cycle 6 is reached with over 66% loss in current density, a trend that is mirrored by a notable increase in the corresponding FE (∼19%). This observed decrease in performance essentially in terms of current density reflects the accumulation of SEI and the resultant increase in interfacial resistance (Figure 4C). Such a decrease in current density has been reported in the literature to pertain to LiNRR, wherein the application of a static cathodic bias tends to accelerate lithium passivation, electrolyte decomposition, and subsequent blockage of the electrode active surface sites.^35^ However, the opposing increase in FE can be traced to the sustained selectivity of previously deposited Li metal toward NRR. The critical transition from productive Li deposition to unproductive Li plating is the predominant factor contributing to the performance degradation observed under elevated overpotentials. The loss of activity and performance across the various cycles (Figure 6A) highlights the inadequacy and limitations of continuous potentiostatic operation in achieving sustained, reliable performance under high current densities over time.

The performance of the NFGDE was also evaluated under alternating cathodic and anodic pulsing, each for 1 min pulse period (Figure 6B). In contrast to constant potentiostatic NFGDE recycling (Figures 6A and S4), the yield of NH_3_ consistently remains lower and did not follow a stable path throughout the 7 cycles of the experiment, specifically in the range of ca. 0.9 mg h^–1^ cm^2^ to 0.2 mg h^–1^ cm^2^, with the FE showing a similar trend with value hitting ∼3% at the end of the seventh cycle. The application of pulsed electrolysis improved electrode longevity by maintaining stable current densities over extended cycling; however, this was accompanied by a decrease in NRR selectivity, likely due to partial oxidation of the electrode–catalyst structure during repeated lithiation and delithiation. The critical importance of dynamic interfacial control within Li-mediated systems as a means to sustain efficient ion transport while suppressing the continuous growth of the SEI comes at the expense of NH_3_ oxidation, electrode, and catalyst oxidation even with a sustained composition after testing (Figure S5) if the oxidative pulse potential is not adequately controlled.

To evaluate the hypothesis that prolonged pulse durations and anodic potentials may induce NH_3_ and electrode-catalyst oxidation while mitigating excessive SEI growth, we applied a pulsing protocol consisting of an anodic pulse at +3 V for 1 s followed by a −12 V cathodic pulse for 10 s (Figure 6C). Despite its initial effectiveness in sustaining a remarkable yield of NH_3_ close to that of the constant potentiostatic case (Figure 6A), there was a gradual decline when subjected to multiple cycles, leading to a reduction in yield from 1.2 to 0.6 mg h^–1^ cm^–2^ after the fifth cycle. Furthermore, the increase in FE to 8.4% indicates a reduced impact of the control on the SEI growth and lithiation–delithiation dynamics, attributable to the lower anodic potential and shorter pulse duration, which also suppressed NH_3_ oxidation. This improvement in selectivity, however, was accompanied by a substantial decrease in current density of ∼62% (Figure 5B). These empirical findings elucidate a nuanced equilibrium that exists between the pulse durations and the corresponding thickness of the SEI (Figure S6). The current density and FEs of previous studies and their comparison with performance metrics in this work are shown in Figure 6D and Table S1.

To study the underlying mechanisms responsible for the NiCu_1_Ru_100_ electrochemical Li-NRR, an in situ Raman test was performed using a customized cell (Figure 7A) to unravel the Li-mediated processes. Prepared NiCu_1_Ru_100_, Ag wire, Pt coil, and 1 M LiBF_4_ with 0.25% ethanol in THF were used as the working electrode, quasi-reference electrode, counter electrode, and electrolyte, respectively. The N_2_ gas was continuously bubbled through the gas inlet to the electrolyte while the in situ Raman test was carried out. The in situ test was performed at a cathodic voltage of −12 V. As shown in Figure 7B, a Li_3_N peak gradually appears and weakens over a 1 h test. The Li_3_N was subsequently protonated, yielding NH_3_, as evidenced by a decrease in the Li_3_N peak signal. Moreover, peaks of lithium intermediates of LiH and LiOH were observed, and this is consistent with the Raman ex situ test (Figure 4A) as well as a THF peak (Figure 7C). More importantly, the active formation of Li_3_N phase is a major step in the effective lithium-mediated NRR process, supporting that the Li^+^ ion is reduced on the NiCu_1_Ru_100_ electrode to activate N_2_ molecules alongside the spontaneous protonation to produce NH_3_. The LiF peak can be traced to the reduction of LiBF_4_ according to eq 4.

4 $${LiBF}_{4}+{3Li}^{+}+{3e}^{-}\overset{}{\to }3LiF+{3Li}_{x}{BF}_{y}$$

Herein, we likewise report the energy input (MWh_Elec._/ton_NH_3__) and the production energy efficiency, PEE (%) for the optimized conditions for the two cell configurations, two important parameters in the evaluation of the performance of the LiNRR system, which is rarely evaluated in most similar systems in the literature. The production energy efficiency (%) was calculated using eq 3. The highest production energy efficiency was 1.37% in the H-cell and corresponds to an electrical energy input of 402.96 MWh/NH_3_ (Table 1). In the case of the single-cell, the production energy efficiency (%) decreases to 0.78% at full cell potential of −12.29 V, mainly due to the significant increase in current density on account of lower ohmic losses and electrode proximity leading to NH_3_ oxidation, which increases the overall cost per ton of NH_3_ at an energy input of 709.52 MWh/NH_3_ (Figure 8). This work establishes a framework for evaluating electrochemical cell architectures to mitigate NH_3_ oxidation, improve ammonia yield, and enhance production energy efficiency in Li-mediated ammonia synthesis systems, while providing important benchmarking metrics relative to the conventional Haber–Bosch process, which typically produces ammonia at approximately $300–$500 per ton depending on natural gas and energy prices (Figure 8).

**Figure 7:**
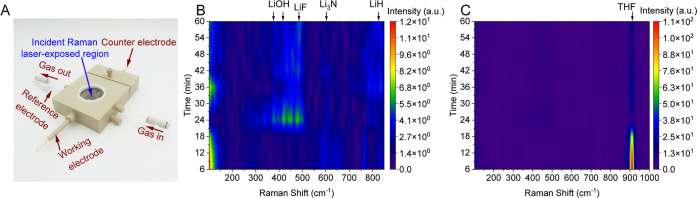
Investigation of the SEI layer via an in situ Raman test of the NiCu_1_Ru_100_ electrode. (A) Schematic setup of the in situ Raman spectra measurement. (B,C) Contour maps of Raman spectra of the NiCu_1_Ru_100_ in the Li-NRR process.

**Table 1:** Performance Metrics Comparison of Two Different Cell Architectures

cell type	full-cell potential (V)	current density (mA cm^–2^)	NH_3_ (μmol)	energy (kJ)	PEE (%)	FE (%)
H-cell	–12.39	–16.3	198.5	4.9	1.37	14.9
single-cell	–12.29	–80.5	123.5	5.3	0.78	8.5

**Figure 8:**
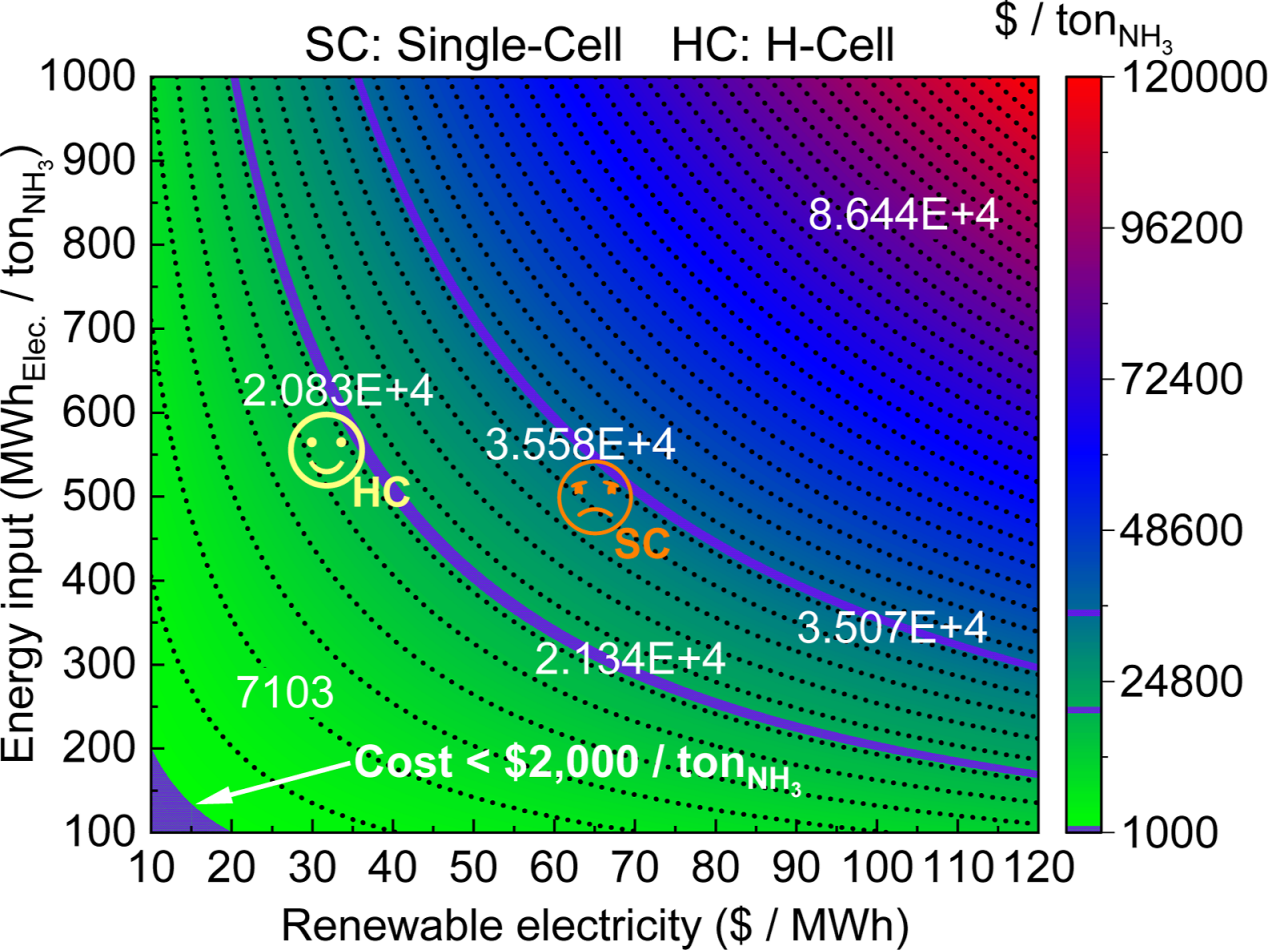
Ammonia production cost as a function of energy consumption and renewable electricity price. The electricity price was assumed constant at $50 per MWh in the cost analysis of the two cell configurations shown in the figure.

The overview of the reaction pathways as supported by the reported catalyst composition (Figure 1), oxidation (Figure 2), activity and selectivity (Figure 3), ex situ SEI properties (Figure 4), electrode recycling cycles (Figure 6), and Li-intermediates via in situ Raman testing (Figure 7) is presented in Figure 9. This overview serves to outline the effect and implications of constant potentiostatic bias and pulse electrolysis alongside the catalytic contribution of the NiCu_1_Ru_100_ electrode toward Li-mediation and the nitrogen reduction reaction for ammonia synthesis.

**Figure 9:**
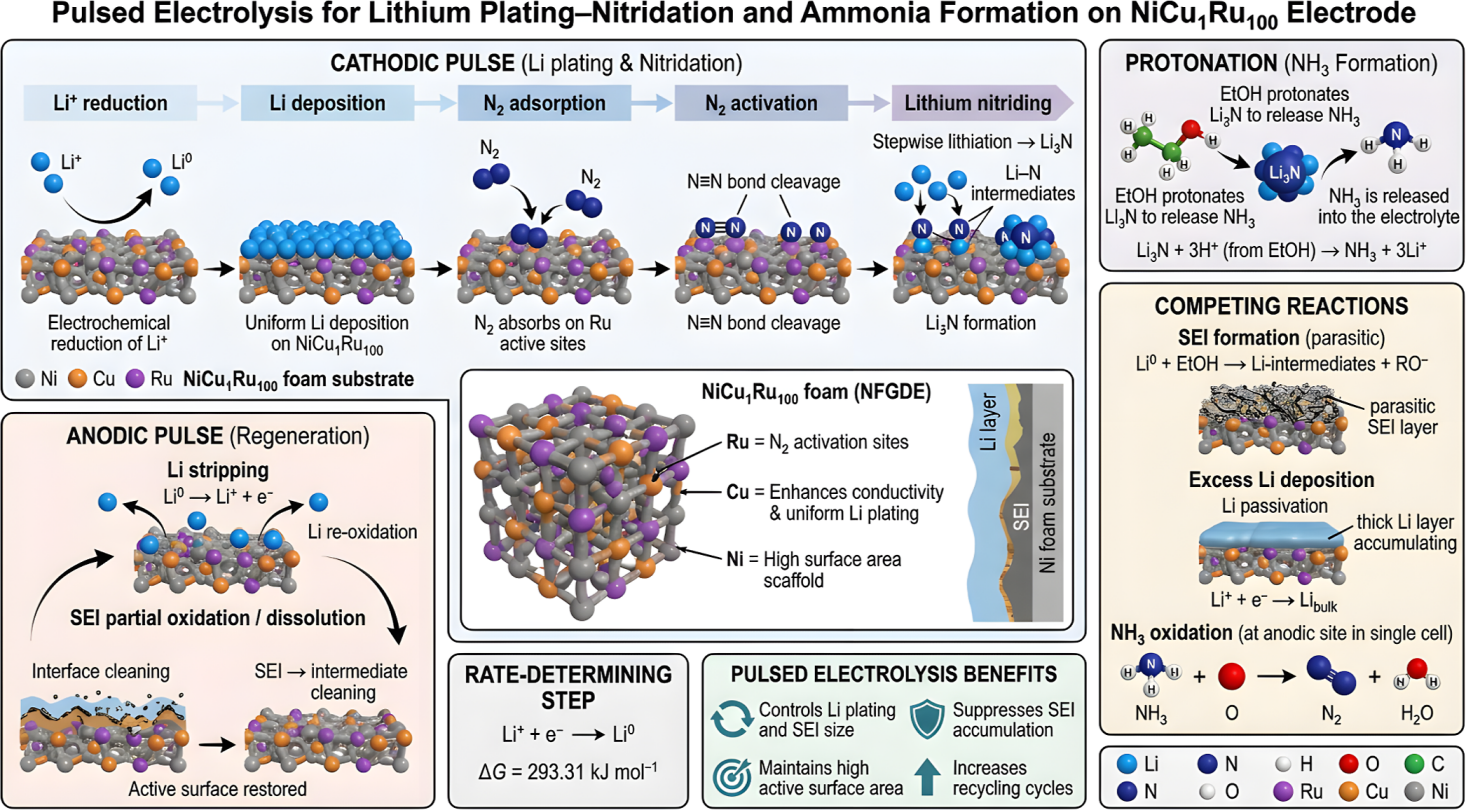
Proposed reaction mechanism pathways during pulsed lithium-mediated electrochemical ammonia synthesis on the NiCu_1_Ru_100_ foam electrode (NFGDE). Schematic illustration of the cathodic and anodic pulse sequence governing lithium plating, nitrogen activation, nitridation, and ammonia generation. During the cathodic pulse, Li^+^ ions are electrochemically reduced and uniformly deposited on the conductive NiCu_1_Ru_100_ foam surface, where Ru active sites facilitate N_2_ adsorption and activation. Progressive lithiation promotes nitrogen triple bond weakening and cleavage, leading to the formation of lithium nitride intermediates through stepwise nitridation. Subsequent protonation by ethanol (EtOH) converts Li_3_N into NH_3_, releasing ammonia into the electrolyte while regenerating Li^+^ species. During the anodic pulse, partial lithium stripping and controlled oxidation/dissolution of the solid electrolyte interphase (SEI) layer restore catalytically active sites, suppress excessive Li accumulation, and regenerate the electrode surface for subsequent reaction cycles. The multifunctional architecture of the NiCu_1_Ru_100_ foam is highlighted, where Ru serves as the primary activation center, Cu enhances electronic conductivity and promotes uniform Li plating, and Ni foam provides a high-surface-area conductive scaffold. The figure also summarizes competing parasitic pathways, including SEI formation due to electrolyte decomposition, excessive Li passivation, and potential NH_3_ oxidation at anodic sites, while illustrating the advantages of pulsed electrolysis for controlling Li deposition, minimizing SEI buildup, maintaining active surface area, and improving cycling stability and NH_3_ production efficiency.

## 4 Conclusions

The NiCu_1_Ru_100_ foam served as a high-surface-area, catalytically active, and durable electrode, enabling stable lithium cycling and efficient nitrogen reduction. Through a comprehensive investigation of a single-cell configuration, we thoroughly examined the critical issue of ammonia crossover, and we addressed this challenge through an innovative approach in cell design and meticulously controlled experiments, which in turn ensured a precise and reliable evaluation of the Faradaic efficiency associated with the process. More significantly, our implementation of pulsed electrolysis was found to control SEI growth and enable prolonged operation across multiple cycles at double-digit current densities. The Faradaic efficiency and the production energy efficiency of the optimized condition for cell geometry between the single and H-cell remain noteworthy (single-cell: 8.5% and 0.78%; H-cell: 14.9% and 1.37%), albeit with current densities of −80.5 mA cm_geo_^–2^ and −16.3 mA cm_geo_^–2^, respectively. We believe that further optimization of the proton and electrolyte salt concentrations can further enhance performance. Furthermore, it was observed that the anodic pulsing potential and pulse durations influence the extent of ammonia oxidation in the single-cell configuration, possibly also affecting the SEI composition and electrode-catalyst oxidation. Therefore, the findings of this work advance the feasibility of decentralized Li-mediated ammonia synthesis at industrially relevant current densities.

## Supplementary Material



## Notes

Scheme for electrode fabrications, ammonia quantification calibration curves, current–time curves for electrochemical Li-NRR in Ar- and N_2_-saturated environments, electrolyte degradation in pulsed electrolysis, SEM and EDX mappings of the cathode after electrolysis, images of electrodes after pulsing (PDF)

## References

[1] Zhou L., Li X., Li Q., Kalu A., Liu C., Liu X., Li W. (2023). Advances in
nitrogen carriers for chemical looping processes for
sustainable and carbon-free ammonia synthesis. ACS Catal..

[2] Duan F., Chen J., Zhang M., Liu Y., Xue H., Sun Y., Li Q., Zhang X., Gao Z., Lu Z. (2025). others Lithium-mediated ammonia electrosynthesis
over orderly arranged
dipoles regulated solid-electrolyte interphase. J. Am. Chem. Soc..

[3] Li K., Shapel S. G., Hochfilzer D., Pedersen J. B., Krempl K., Andersen S. Z., Sazinas R., Saccoccio M., Li S., Chakraborty D. (2021). others Increasing current density of Li-mediated
ammonia synthesis with high surface area copper electrodes. ACS Energy Lett..

[4] Tort R., Westhead O., Spry M., Davies B. J., Ryan M. P., Titirici M.-M., Stephens I. E. (2023). Nonaqueous
Li-mediated nitrogen reduction:
taking control of potentials. ACS Energy Lett..

[5] Andersen S. Z., Čolić V., Yang S., Schwalbe J. A., Nielander A. C., McEnaney J. M., Enemark-Rasmussen K., Baker J. G., Singh A. R., Rohr B. A. (2019). others A rigorous electrochemical ammonia synthesis
protocol with quantitative isotope measurements. Nature.

[6] Iriawan H., Herzog A., Yu S., Ceribelli N., Shao-Horn Y. (2024). Upshifting lithium plating potential
to enhance electrochemical
lithium mediated ammonia synthesis. ACS Energy
Lett..

[7] Huang H., Tu W., Fang L., Xiao Y., Niu F., Zhu H., Zhu X., Wang L., Xiong Y., Feng J. (2023). others
Lithium-mediated photoelectrochemical ammonia synthesis with 95% selectivity
on silicon photocathode. ACS Energy Lett..

[8] Fu X., Li S., Deissler N. H., Mygind J. B. V., Kibsgaard J., Chorkendorff I. (2024). Effect of
lithium salt on lithium-mediated ammonia
synthesis. ACS Energy Lett..

[9] Hodgetts R. Y., Du H.-L., Nguyen T. D., MacFarlane D., Simonov A. N. (2022). Electrocatalytic oxidation of hydrogen as an anode
reaction for the Li-mediated N2 reduction to ammonia. ACS Catal..

[10] Jeong Y., Janani G., Kim D., An T.-Y., Surendran S., Lee H., Moon D. J., Kim J. Y., Han M.-K., Sim U. (2023). Roles of Heterojunction
and Cu Vacancies in the Au@ Cu2–x Se for the Enhancement of
Electrochemical Nitrogen Reduction Performance. ACS Appl. Mater. Interfaces.

[11] Spry M., Westhead O., Tort R., Moss B., Katayama Y., Titirici M.-M., Stephens I. E., Bagger A. (2023). Water increases the
faradaic selectivity of Li-mediated nitrogen reduction. ACS Energy Lett..

[12] Bi W., Bao W., Gyenge E. L., Wilkinson D. P. (2025). Lithium-Mediated Nitrogen Reduction
in a Flow Electrolyzer Cell Using a Gas-Diffusion Cathode with Carbonaceous
Reaction Layers. ACS Appl. Energy Mater..

[13] Burdis C., Tort R., Winiwarter A., Rietbrock J., Barrio J., Titirici M. M., Stephens I. E. (2025). A carbon
cathode
for lithium mediated electrochemical ammonia synthesis. Energy Environ. Sci..

[14] Westhead O., Spry M., Bagger A., Shen Z., Yadegari H., Favero S., Tort R., Titirici M., Ryan M. P., Jervis R. (2023). others
The role of ion solvation in lithium mediated
nitrogen reduction. J. Mater. Chem. A.

[15] Han Y., Lim C., Kim Y., Baek H., Jeon S., Han J. W., Yong K. (2024). Steric Hindrance-Derived Li+ Solvation to Enhance Lithium-Mediated
Nitrogen Reduction. ACS Energy Lett..

[16] Lim C., Kim D., Kim M., Yun H., Shin D., Hwang Y. J., Shin H., Yong K. (2023). Effect of
sulfur-derived solid electrolyte
interphase on Li-mediated nitrogen reduction. ACS Energy Lett..

[17] Feng S., Gao W., Guo J., Cao H., Chen P. (2023). Electrodriven chemical
looping ammonia synthesis mediated by lithium imide. ACS Energy Lett..

[18] Xu J., Liu Y., Zhao W., Lv X., Yang X., Zhao X. (2025). Optimizing
Interface Permeability and Structural Stability through a Fine Inorganic-Grain-Network
for Long-Term Lithium-Mediated Ammonia Synthesis. ACS Sustainable Chem. Eng..

[19] Lim C., Sohn Y., Kwon M., Seo D.-H., Kim W., Yong K. (2025). Suppressing
Anion Repulsion for Enhancing Li-Mediated Nitrogen Reduction. ACS Energy Lett..

[20] Lazouski N., Schiffer Z. J., Williams K., Manthiram K. (2019). Understanding
continuous lithium-mediated electrochemical nitrogen reduction. Joule.

[21] Kim M., Kim J., Yun H., Jeon Y., Hwang Y. J., Yong K. (2026). Advancing
Lithium-Mediated Nitrogen Reduction using Insights from Lithium-Ion
Battery: Focusing on Uniform Li Plating. Adv.
Energy Mater..

[22] Montoya J. H., Tsai C., Vojvodic A., Nørskov J. K. (2015). The challenge
of electrochemical ammonia synthesis: a new perspective on the role
of nitrogen scaling relations. ChemSusChem.

[23] Boppella R., Ahmadi M., Arndt B. M., Lustig D. R., Nazemi M. (2024). Pulsed electrolysis
in membrane electrode assembly architecture for enhanced electrochemical
nitrate reduction reaction to ammonia. ACS Catal..

[24] Andersen S. Z., Statt M. J., Bukas V. J., Shapel S. G., Pedersen J. B., Krempl K., Saccoccio M., Chakraborty D., Kibsgaard J., Vesborg P. C. (2020). others
Increasing stability,
efficiency, and fundamental understanding of lithium-mediated electrochemical
nitrogen reduction. Energy Environ. Sci..

[25] Jin D., Chen A., Lin B.-L. (2024). What metals should be used to mediate
electrosynthesis of ammonia from nitrogen and hydrogen from a thermodynamic
standpoint?. J. Am. Chem. Soc..

[26] Kolen M., Ripepi D., Smith W. A., Burdyny T., Mulder F. M. (2022). Overcoming
nitrogen reduction to ammonia detection challenges: the case for leapfrogging
to gas diffusion electrode platforms. ACS Catal..

[27] Yang S., Sohn Y., Chu J., Kim W., Shin B. (2025). Unveiling
the Mechanisms of Improved Stability and Performance via Tetraalkyl-Type
Ionic Liquids: Suppression of Organic Solid Electrolyte Interface
Formation in Lithium-Mediated Nitrogen Reduction. ACS Appl. Mater. Interfaces.

[28] Sazinas R., Li K., Andersen S. Z., Saccoccio M., Li S., Pedersen J. B., Kibsgaard J., Vesborg P. C., Chakraborty D., Chorkendorff I. (2022). Oxygen-enhanced chemical stability of lithium-mediated
electrochemical ammonia synthesis. J. Phys.
Chem. Lett..

[29] Wu Y., Zhang M., Zhao Z., Song W., Yan J., Wen J., Mahmood
Ali A., Ge X., Zhang H. (2025). Recent Progress and
Future Outlook on Catalysts for Ammonia Electrosynthesis: Materials,
Structural Design, and Reaction Efficiency. Energy Fuel.

[30] Li J., Zhan G., Yang J., Quan F., Mao C., Liu Y., Wang B., Lei F., Li L., Chan A. W. (2020). others Efficient ammonia
electrosynthesis from nitrate on strained
ruthenium nanoclusters. J. Am. Chem. Soc..

[31] Zhang J., Li G., Guo J., Fan H., Chen P., Jiang L., Xie H. (2022). Spectroscopic Characterization
of the Synergistic Mechanism of Ruthenium–Lithium
Hydrides for Dinitrogen Cleavage. J. Phys. Chem.
Lett..

[32] Kou Z., Shi D., Yang B., Li Z., Zhang Q., Lu J., Zhang T., Lei L., Li Y., Dai L. (2025). others Efficient green synthesis of ammonia:
from mechanistic understanding
to reactor design for potential production. Chem. Soc. Rev..

[33] Turner C., Španěl P., Smith D. (2006). A longitudinal study of ammonia,
acetone and propanol in the exhaled breath of 30 subjects using selected
ion flow tube mass spectrometry, SIFT-MS. Physiol.
Meas..

[34] Schlesinger W. H., Hartley A. E. (1992). A global budget
for atmospheric NH3. Biogeochemistry.

[35] Cherepanov P. V., Krebsz M., Hodgetts R. Y., Simonov A. N., MacFarlane D. R. (2021). Understanding
the factors determining the faradaic efficiency and rate of the lithium
redox-mediated N2 reduction to ammonia. J. Phys.
Chem. C.

[36] Kani N. C., Goyal I., Gauthier J. A., Shields W., Shields M., Singh M. R. (2024). Pathway toward scalable
energy-efficient Li-mediated
ammonia synthesis. ACS Appl. Mater. Interfaces.

[37] Cheu D., Adams T., Revankar S., Pol V. (2024). Evaluation
of lithium
as a tritium storage medium for betavoltaics. J. Appl. Phys..

[38] Mangini A., Garbujo A., Biasi P., Testa V., Bruzzoniti M. C., Rivoira L., Garcia-Ballesteros S., Bella F. (2025). Debunking Pitfalls
of Li–N2 Cells for Ammonia Electroproduction: Is This Setup
Affordable to Prove Nitro-Fixation before Lithium Plating?. ACS Electrochem..

[39] Westhead O., Tort R., Spry M., Rietbrock J., Jervis R., Grimaud A., Bagger A., Stephens I. L. (2023). The origin
of overpotential in lithium-mediated nitrogen reduction. Faraday Discuss..

[40] Lazouski N., Steinberg K. J., Gala M. L., Krishnamurthy D., Viswanathan V., Manthiram K. (2022). Proton donors induce a differential
transport effect for selectivity toward ammonia in lithium-mediated
nitrogen reduction. ACS Catal..

[41] Deissler N. H., Mygind J. B. V., Li K., Niemann V. A., Benedek P., Vinci V., Li S., Fu X., Vesborg P. C., Jaramillo T. F. (2024). others Operando investigations
of the solid
electrolyte interphase in the lithium mediated nitrogen reduction
reaction. Energy Environ. Sci..

