# Supplementary material for: Improving the Longevity of Li-Mediated Ammonia Synthesis via Pulsed Electrolysis under High Current Densities
Source: ACS Appl Mater Interfaces. 2026 Jun 2;18(23):32616–29. doi: 10.1021/acsami.6c04372 (PMC13288392; doi:10.1021/acsami.6c04372)
Supplement: Supplementary file 1 [file am6c04372_si_001.pdf]

SUPPORTING INFORMATION
Improving the Longevity of Li-Mediated Ammonia Synthesis via Pulsed Electrolysis under High Current Densities

Ojo Friday Abraham[a], Brenden M. Arndt[a], and Reza Nazemi[a,b*]

[a]School of Materials Science and Engineering, Colorado State University, Fort Collins, Colorado 80524, United States

[b]Department of Mechanical Engineering, Colorado State University, Fort Collins, Colorado 80523, United States

Email Address: Reza.Nazemi@colostate.edu

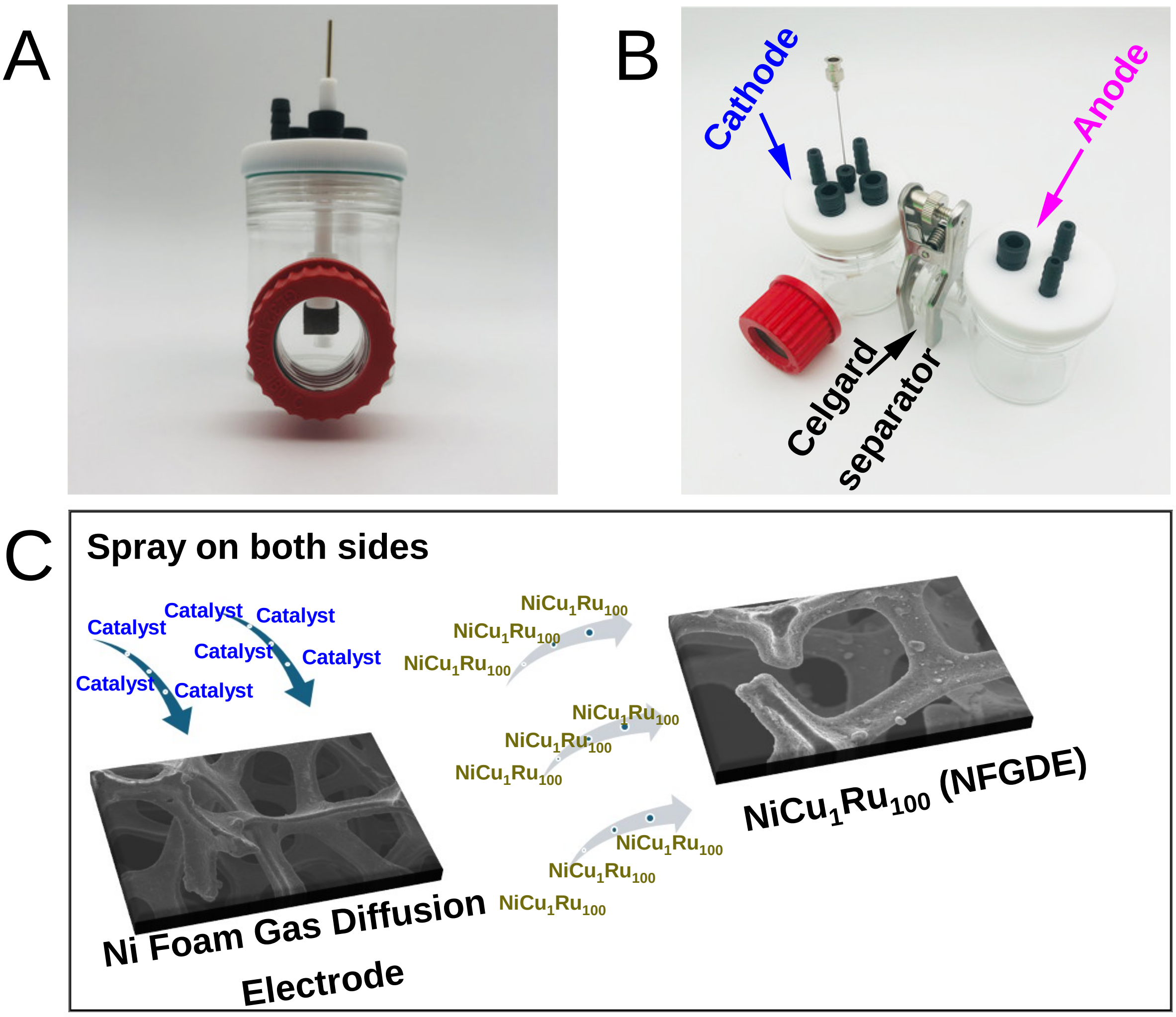

Fig. S1: A) Single-cell and B) H-cell configurations and C) Scheme for the fabrication of $NiCu_1Ru_{100}$ electrodes.

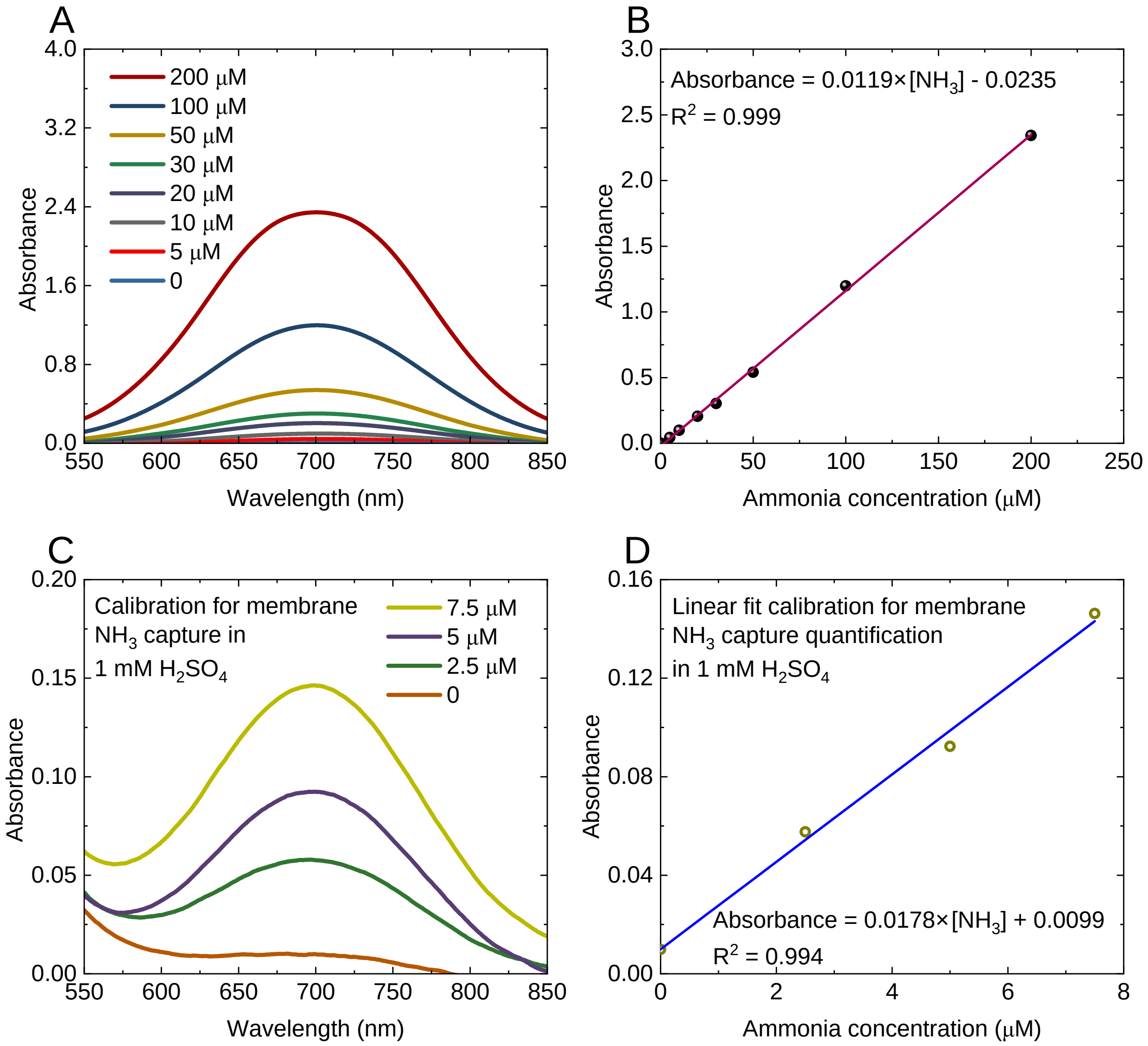

Fig. S2: Calibration curves for NH_3 quantification using modified indophenol method. **A**) Absorbance spectra containing 10% of 1M LiBF4 + 0.25% EtOH in THF + 90% water with known ammonia concentrations. **B**) Calibration curve from the absorbance spectra at 697 nm in A. **C**) Absorbance spectra containing 1 mM H_2SO_4 and known ammonia concentrations. **D**) Calibration curve from the absorbance spectra at 697 nm in C.

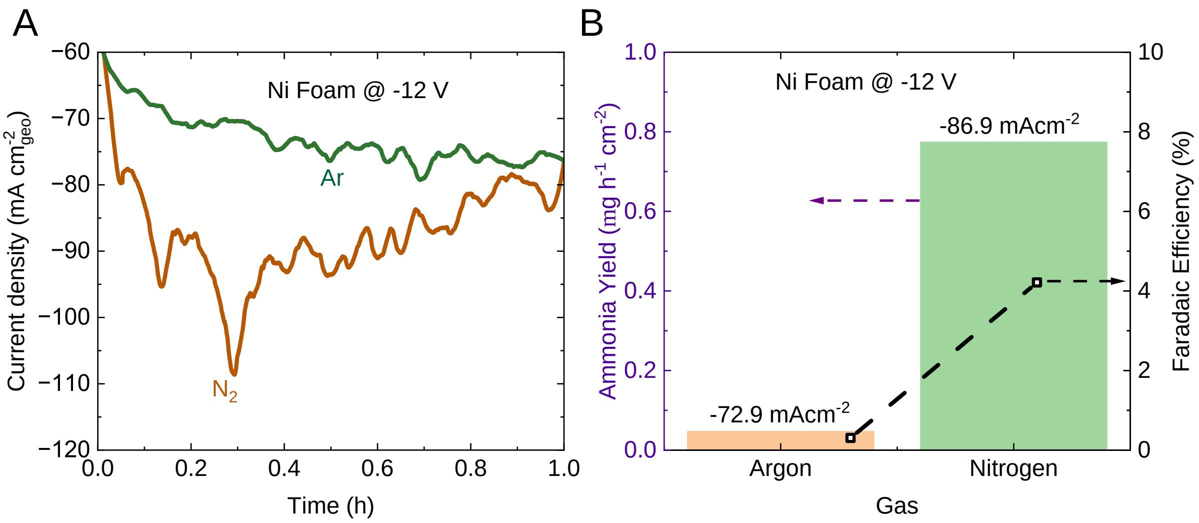

Fig. S3: **A**) Electrochemical Li-NRR current-time curves of Ni foam cathode under saturated Ar and N_2 gas and **B**) NH_3 yield rates and Faradaic efficiencies of electrocatalytic Li-NRR using the Ni foam cathode under saturated Ar and N_2 gas.

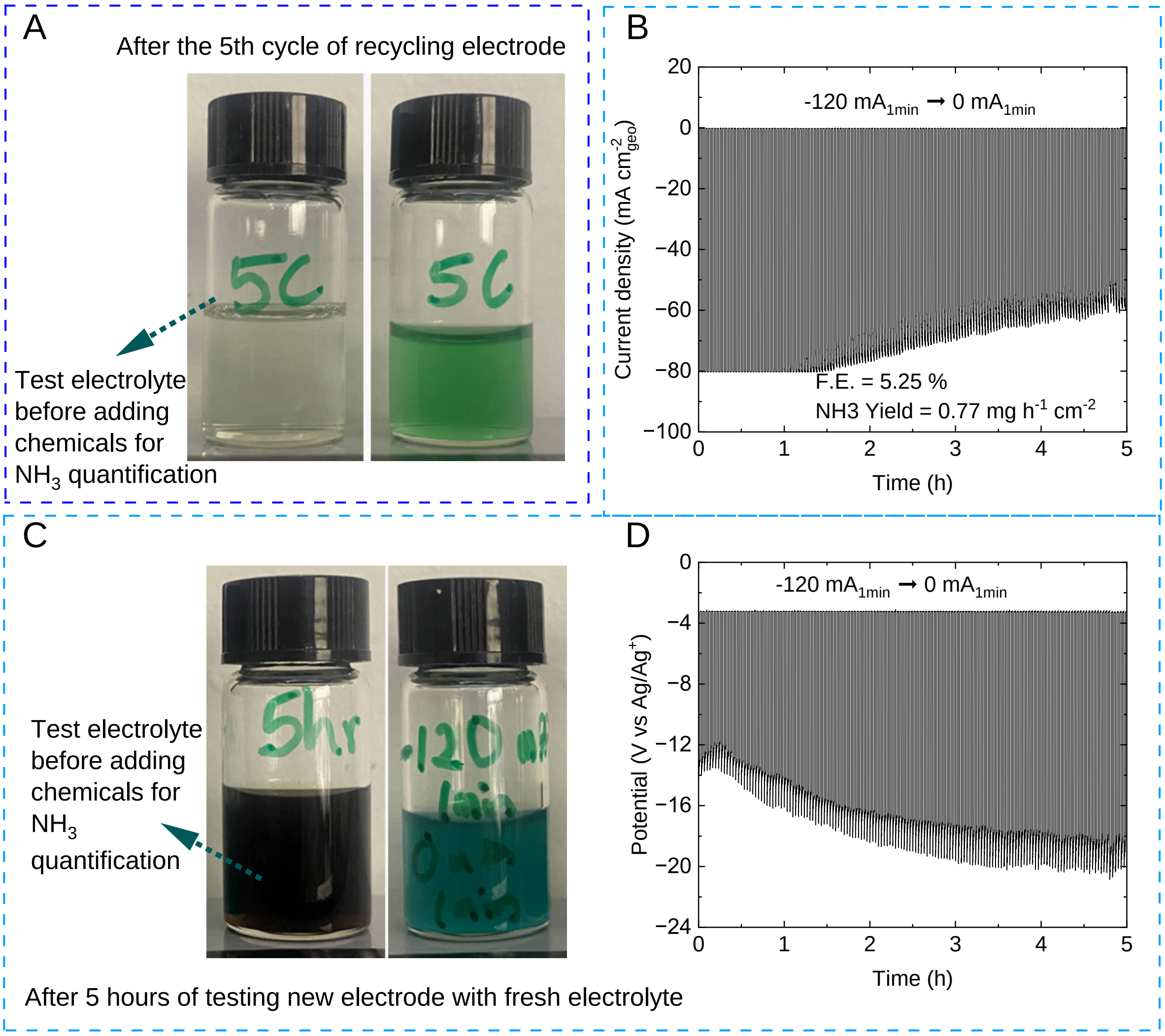

Fig. S4: Electrolyte degradation in various stability testing. **A**) Pulsing between cathodic and anodic potentials for cathode recycling after the 5th cycle (equivalent to 5 hours of electrode recycling), **B**) pulsing only in the cathodic region under -120 mA for 1 minute to 0 mA for 1 minute for a total time of 5 hours, **C**) Electrolyte condition under pulsing in only the cathodic region under -120 mA for 1 minute to 0 mA for 1 minute for a total time of 5 hours, and **D**) Potential response following the current pulsing in B).

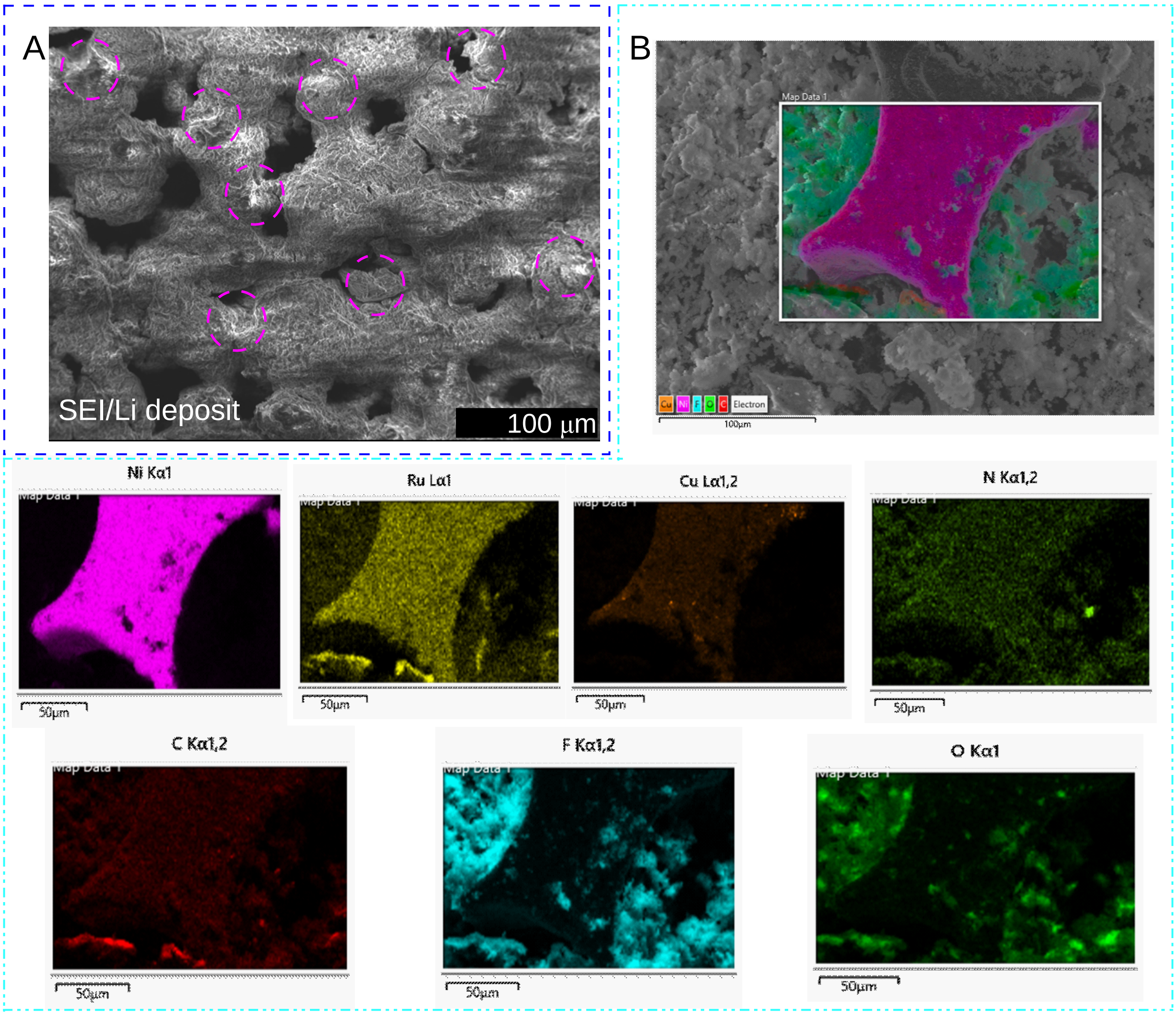

Fig. S5: **A**) SEM image of $NiCu_1Ru_{100}$ after electrochemical Li-NRR, and **B**) EDX elemental mappings of synthesized catalyst, Cu_1Ru_{100}, spray-coated ($1mg/cm^2$) on nickel foam after Li-NRR.

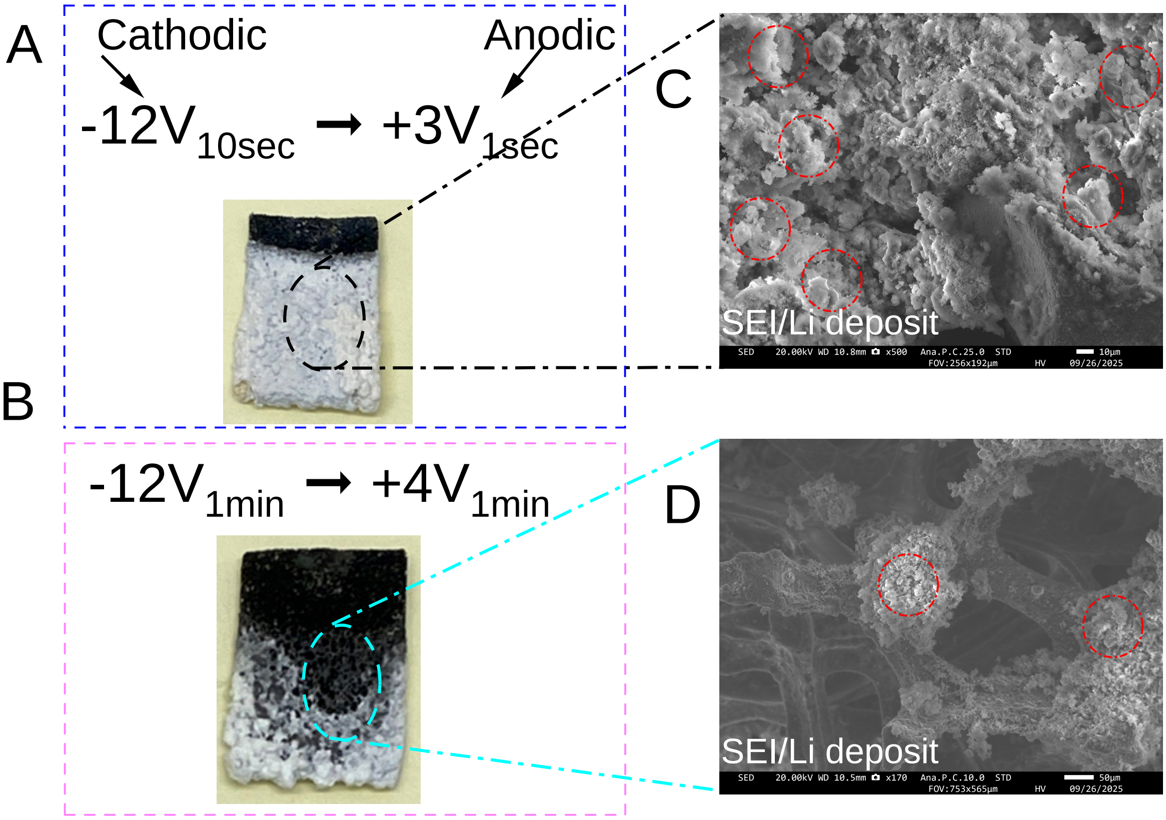

Fig. S6: Visual view of electrodes after pulsing under cathodic and anodic potentials for **A**) cathodic bias at 10 seconds and anodic bias for 1 second, **B**) cathodic bias at 1 minute (60 seconds) and anodic bias for 1 minute (60 seconds) and SEM images of electrodes after **C**) cathodic bias at 10 seconds and anodic bias for 1 second, **D**) cathodic bias at 1 minute (60 seconds) and anodic bias for 1 minute (60 seconds)

Table S1: Comparison of electrochemical LiNRR reported in the literature

Cathode materials	Electrolyte composition	Current density (mA cm^{-2}_{geo})	Applied pressure (bar)	Ammonia yield rate (nmol s^{-1} cm^{-2}_{geo})	Faradaic efficiency (%)	Ref.
$NiCu_1Ru_{100}$	1 M $LiBF_4$ in THF (0.25% EtOH)	-80.51	1	23.68	8.50	**This work**
$NiCu_1Ru_{100}$ (after 6th cycle)	1 M $LiBF_4$ in THF (0.25% EtOH)	-33.01	1	21.58	18.92	**This work**
$BaTiO_3$	0.2 M $LiBF_4$ in THF (2% EtOH)	-0.75	1	6.94	93.00	[1]
Copper foil	1 M $LiBF_4$ in THF (0.1 M EtOH)	-8	1	5.12	18.54	[2]
HBTCu	2 M $LiClO_4$ in THF (1% EtOH)	-100	20	46	13.30	[3]
Si wafer	0.2 M $LiBF_4$ in THF (1% EtOH)	-0.25	1	0.85	95.00	[4]
Mo foil	0.6 M $LiClO_4$ in THF (1% EtOH)	-2	1	60	7.8	[5]

References

[1] Fangying Duan, Junwu Chen, Mengfei Zhang, Yiming Liu, Hao Xue, Yu Sun, Qiongguang Li, Xuehua Zhang, Zijian Gao, Zongjing Lu, Schwaller Philippe, Guangjin Zhang, and Jian Zhan. Lithium-mediated ammonia electrosynthesis over orderly arranged dipoles regulated solid-electrolyte interphase. *Journal of the American Chemical Society*, 2025.

[2] Nikifar Lazouski, Zachary J Schiffer, Kindle Williams, and Karthish Manthiram. Understanding continuous lithium-mediated electrochemical nitrogen reduction. *Joule*, 3(4):1127–1139, 2019.

[3] Katja Li, Sarah G Shapel, Degenhart Hochfilzer, Jakob B Pedersen, Kevin Krempl, Suzanne Z Andersen, Rokas Sazinas, Mattia Saccoccio, Shaofeng Li, Debasish Chakraborty, et al. Increasing current density of li-mediated ammonia synthesis with high surface area copper electrodes. *ACS Energy Letters*, 7(1):36–41, 2021.

[4] Hao Huang, Wenguang Tu, Liping Fang, Yelan Xiao, Feng Niu, Heng Zhu, Xi Zhu, Lu Wang, Yujie Xiong, Jianyong Feng, et al. Lithium-mediated photoelectrochemical ammonia synthesis with 95% selectivity on silicon photocathode. *ACS Energy Letters*, 8(10):4235–4241, 2023.

[5] Olivia Westhead, Matthew Spry, Alexander Bagger, Zonghao Shen, Hossein Yadegari, Silvia Favero, Romain Tort, M Titirici, Mary P Ryan, Rhodri Jervis, et al. The role of ion solvation in lithium mediated nitrogen reduction. *Journal of Materials Chemistry A*, 11(24):12746–12758, 2023.
